# Influence of Off-Centre Positioning, Scan Direction, and Localiser Projection Angle on Organ-Specific Radiation Doses in Low-Dose Chest CT: A Simulation Study Across Four Scanner Models

**DOI:** 10.3390/jimaging12030123

**Published:** 2026-03-11

**Authors:** Louise D’hondt, Claudia Haentjens, Pieter-Jan Kellens, Annemiek Snoeckx, Klaus Bacher

**Affiliations:** 1 Department of Human Structure and Repair, Faculty of Medicine and Health Sciences, Ghent University, 9000 Ghent, Belgium; pjkellens@icloud.com (P.-J.K.); klaus.bacher@ugent.be (K.B.); 2 Faculty of Medicine, University of Antwerp, 2610 Wilrijk, Belgium; annemiek.snoeckx@uza.be; 3 Department of Physics and Astronomy, Faculty of Sciences, Ghent University, 9000 Ghent, Belgium; claudiahaentjens@gmail.com; 4 Department of Radiology, Antwerp University Hospital, 2650 Edegem, Belgium

**Keywords:** low-dose computed tomography, radiation dosimetry, Monte Carlo simulations, automatic tube current modulation, off-centre positioning, localiser radiograph, scan direction, lung cancer screening

## Abstract

With the considerable number of low-dose CT examinations performed in lung cancer screening, variations in participant positioning, scan direction, or localiser angle are likely to occur in practice. These variations are known to affect automatic tube current modulation (ATCM) operation, yet organ-specific dose implications across CT models remain unknown. Therefore, this simulation study systematically characterised the effect of the aforementioned variations. Using the Alderson RANDO phantom, ATCM profiles were established on CT scanners from four major vendors (GE, Siemens, Canon, Philips) after introducing vertical and lateral mispositioning, craniocaudal and caudocranial scan directions, and varying localiser projection angles. Additionally, off-centre positioning and scan direction changes preceded by either a single posteroanterior (PA) or dual (PA+lateral) localiser were evaluated. Doses to the lungs, heart, thyroid, liver, and breasts were calculated from Monte Carlo simulations of each setup for 32 patient-specific voxel models. The results demonstrate statistically significant and scanner-dependent dose variations. PA localisers generally produced the highest organ doses. However, on the Philips system, organ dose increases of at least 50% were observed after the lateral projection angle. GE and Siemens scanners showed pronounced dose increases following downward mispositioning with a single PA localiser (18–50% and 5–25%, respectively), an effect largely mitigated by adding a lateral localiser. Canon and Philips scanners exhibited generally stable ATCM behaviour after vertical off-centring, although Canon showed notable dose increases upon lateral mispositioning, with dose increases up to 37.5% and 34% after a single PA or dual localiser, respectively. Variations in scan direction displayed highly model- and organ-dependent effects. Dose deviations were largely mitigated after dual localisers for the GE, Canon, and Philips scanner types. Here, organ dose differences were within an absolute range of 10%, indicating that a change in scan direction preceded by a dual localiser can reduce extreme dose deviations. Remarkably, no significant difference was observed solely for the Siemens scanner when combined with a dual localiser, as lung, heart, breast, and liver doses remained significantly (between 20 and 35%) lower when scanning craniocaudally, whereas the thyroid dose in this setup remained considerably higher (up to 20% mean increase). Ultimately, findings indicate that seemingly minor protocol deviations can lead to significant underestimation of anticipated organ-specific doses associated with lung cancer screening. Scanner-specific optimisation, supported by medical physics expertise, is therefore essential.

## 1. Introduction

The increasing number of computed tomography (CT) examinations over the last few decades substantially contributes to the radiation dose from medical imaging to the population [1,2,3,4,5,6,7,8,9]. As CT renders a relatively high radiation burden compared to conventional radiographic examinations, automatic exposure control (AEC) technologies were developed to optimise radiation dose reductions across different-sized patients while improving the consistency of image quality [4,6,7,10,11,12,13,14]. One of the most useful developments is automatic tube current modulation (ATCM), which has become a standard technological feature on all modern CT scanners from every major manufacturer [6,7,15]. Based on a two-dimensional localiser radiograph (also named scout, topogram, or scanogram), an estimate of patient size and regional attenuation in the scan range is derived to adapt the tube current, which is required to achieve a predetermined level of image noise [11,12,13,16,17,18,19,20,21]. ATCM systems modulate tube current by means of two processes: z-modulation modifies the X-ray tube current along the longitudinal axis of the scanned patient, while xy- or angular modulation changes the radiation output as a function of the projection angle during rotation around the patient [4,8,14,20].

While it has been proven that ATCM systems have the potential to significantly reduce the dose of CT examinations [4,7,14,20], the exact underlying mechanisms of modulation vary between scanners and are mostly proprietary to different manufacturers [11,16,18,22,23,24,25,26]. Numerous studies have investigated these mechanisms and have shown that the radiation output of the diagnostic scan itself is influenced by the preceding localiser acquisition [10,11,12,15,16,17,19,21,22,23,24,27,28,29,30,31]. It has been demonstrated by these studies that the ATCM definition varies by the angle at which the localiser radiograph is taken, especially in combination with inaccurate patient positioning. When the patient is not accurately centred within the CT scanner gantry, geometric magnification or minification of patient structures on the localiser image impairs the working mechanism of ATCM systems and, as such, negatively impacts radiation dose and image quality [3,9,11,15,16,18,21,22,28,29,32,33,34,35,36,37,38,39,40,41,42]. The significant dose increases due to suboptimal ATCM functioning found in the studies can be particularly important, since inaccurate positioning of patients by multiple centimetres is reported to be very common in clinical practice, due to, among others, limited training or experience, high workloads, or patient-specific factors [3,11,28,40,41,43,44].

A drawback of most published studies on the influence of the localiser angle, patient off-centring, or combinations of both is that differences in radiation doses are mostly reported in terms of volume computed tomography dose index (CTDI_vol_). In addition, some studies use technical water-based and/or plastic phantoms that are intended for routine quality assurance and lack realism [5,7,10,11,12,15,16,17,18,19,21,22,23,28,29,30,31,33,39]. While CTDI_vol_ metrics quantify the radiation output relative to a reference protocol, they do not account for patient-specific sizes and structures. The CTDI_vol_ provides an average dose summary estimate of the scanned length, but it does not reflect the changes in exposure due to the ATCM across different organs and body regions for individual patients, even when an anthropomorphic phantom is used [3,19,27,31,40]. Recent studies have applied Monte Carlo simulations to estimate the effect of the CT scanner output in terms of effective dose or absorbed doses per organ [8,9,14,27,38]. Although these studies do consider the distribution of the dose across different organs, the input models for the simulation are still based on phantom models or do not represent a broad clinical representation of actual patients. Finally, while many studies have investigated ATCM behaviour irrespective of the metric as a function of localiser angle, relatively few have considered and quantified a possible effect of the change in scanning direction on the ATCM output [6,24,26,45,46].

In the context of lung cancer screening, where large numbers of asymptomatic participants undergo low-dose chest CT examinations, the potential dose-reducing benefits of ATCM systems could be particularly relevant. Considering that the implementation of screening often entails a substantial additional workload, the risk of off-centre positioning correspondingly increases [3,32,34,44]. As previously mentioned, numerous studies have undoubtedly indicated that the choice of, or accidental variations in, the localiser protocol strongly dictate ATCM performance and, hence, the radiation dose of the subsequent scan, particularly when accompanied by improper positioning. Together, these factors increase the likelihood that participant radiation exposure during low-dose screening CT examinations is not as low as anticipated. However, there is currently no consensus or effective (screening) guidelines on how to optimise the localiser protocol preceding a CT examination. As the localiser acquisition is associated with a considerably smaller dose compared to the diagnostic CT scan, it is less prioritised to be optimised [4,10,12,15,16,17,47]. Some studies suggest using a single posteroanterior (PA) instead of an anteroposterior (AP) projection to limit direct radiation dose to the breast, thyroid, and other sensitive anteriorly situated organs [15,17]. However, this relatively minor dose reduction may be short-sighted, as studies have shown that operators tend to off-centre position patients below the isocentre, resulting in unintended dose increases due to suboptimal ATCM functioning during the diagnostic scan [15,36,41]. Others have suggested using two orthogonally acquired localiser radiographs, debating the enhanced functionality of ATCM over a single radiograph, and improved estimation of patient misalignment [3,12,17,30,39]. Yet for (ultra-)low-dose lung cancer screening protocols, where the relative contribution of the localiser dose is a proportionally larger part of the total effective participant dose, the dose from the additional lateral scout may not be negligible [10,31,48]. As reported by the European Study on Clinical Diagnostic Reference Levels, the median doses (CTDI_vol_) for a standard, diagnostic chest CT are 3.5 mGy for Europe and 8.8 mGy for the United States [49]. In comparison, European screening guidelines recommend striving for CT doses of 0.4 mGy, 0.8 mGy, and 1.6 mGy (for participants <50, 50–80, and >80 kg, respectively) [50]. When the dose from one localiser projection is typically in the range of 0.1 to 0.2 mGy [48], the additional lateral localiser could potentially be considered to be omitted as a dose-saving measure in the context of screening. Therefore, scanner-specific research on the potential influence of using a dual localiser on the ATCM system and subsequent organ-specific doses is required.

The goal of this study was to extensively characterise how varying localiser and scan settings affect ATCM behaviour and, most importantly, subsequent organ-specific radiation doses. By means of an anthropomorphic phantom, we derived ATCM profiles after systematic variations in CT acquisition setup on four different CT scanners, including one scanner of each of four major manufacturers (GE Healthcare, Waukesha, WI, USA; Siemens Healthineers, Erlangen, Germany; Canon Medical Systems, Ōtawara, Japan; and Philips Healthcare, Best, The Netherlands). The systematic variations included variation in localiser projection angle, CT scan direction, and vertical and horizontal patient off-centring. As an additional research objective in the context of lung cancer screening, we investigated whether changes in ATCM profiles upon scan direction variation and off-centring differed when preceded by a single or dual localiser. Translation of experimental phantom-derived ATCM profiles into Monte Carlo simulation software (ImpactMC version 1.2.6, CT imaging GmbH, Erlangen, Germany)enables standardised evaluation of these localiser/scan variations in terms of organ-specific absorbed doses across a broad sample of patient-derived 3D models representing both sexes and four BMI categories.

## 2. Materials and Methods

### 2.1. CT Acquisitions of the Anthropomorphic Phantom

The Alderson RANDO phantom (Radiology Support Devices Inc., Long Beach, CA, USA) was used to experimentally derive ATCM curves for different setups on multiple CT scanners. This anthropomorphic phantom represents an adult male patient and encompasses tissue-equivalent substitutes for the heart, lung, liver, bones, muscle, and adipose tissue [51]. The 2.5 cm axial cross-sectional slices from the head to mid-abdomen were connected. All acquisition setups were performed on the Revolution CT (GE Healthcare, Waukesha, WI, USA), the SOMATOM Definition Flash (Siemens Healthineers, Erlangen, Germany), the Aquilion ONE (Canon Medical Systems, Ōtawara, Japan), and the Spectral CT 7500 (Philips Healthcare, Best, The Netherlands).

On all scanners, we acquired a reference scan. For this, the scanner’s built-in lasers, anatomical landmarks, and annotations on the phantom were used to align the geometric centre with the centre of the CT gantry. The table height coordinates were recorded. A localiser was taken from the PA angle and in the craniocaudal direction, followed by a chest examination. CT scan parameters of the actual diagnostic scan were set to simulate a low-dose CT scan for lung cancer screening, and approximated those defined by the technical standards of the European Society of Thoracic Imaging (ESTI) [50,52]. The CT scanners were operated at a tube voltage of 100 kVp, collimation of 40 mm (GE Revolution CT: 80 mm), and rotation time of 0.5 s (Siemens SOMATOM Definition Flash: 0.6 s); the pitch was scanner-specific, varying from 1.35 to 1.53. The phantom was scanned in helical mode and in caudocranial direction.

In a first series of setups, we investigated ATCM behaviour upon changing the angle at which the localiser was acquired. Instead of a single localiser from the bottom (PA), we acquired acquisitions from top only (AP), lateral only (LAT), or combinations (PA+LAT or AP+LAT). For GE, Siemens, and Canon scanners, these four additional localiser configurations were tested relative to the reference PA localiser. In contrast to the other scanner models, only a single LAT localiser projection angle and PA+LAT combination were possible in addition to the reference on the Philips CT scanner. After the scanogram, all scan parameters remained identical.

Second, we assessed the influence of scan direction. We performed a PA localiser in the craniocaudal direction and, instead of performing the diagnostic scan in the returning caudocranial direction, we started from the lung apices and performed the helical scan in craniocaudal direction. To investigate whether a dual localiser could have an interaction effect with the change in scan direction, the latter setup was also performed, preceded by a PA+LAT localiser. Other scan parameters remained constant.

Finally, we evaluated ATCM profiles with off-centring within the gantry. Along the vertical axis, the phantom was positioned off-centre, either 2, 4, or 6 cm below (−) or above (+) ideal isocentre positioning. This was performed by adjusting the table height with respect to the reference coordinates. For horizontal off-centring, the phantom was shifted 2, 4, or 6 cm to the phantom’s left-hand (−) or right-hand (+) side with respect to the centreline. The horizontal positions were approximated as the phantom was moved manually with respect to the applied marks. At each vertical and lateral deviation, a localiser was acquired initially from the PA angle, and the helical scan was performed as described above. In addition, to assess whether a dual scout could influence ATCM behaviour in combination with non-ideal positioning, a PA+LAT localiser was also acquired at each position deviation, followed by a chest CT.

Note that, in every setup, the phantom was positioned supine, and all scan series were performed with identical scan coverage, ranging from the lung apex to the lung base. From the reference scans and all varying setups, tube current values for each slice were extracted from the Digital Imaging and Communications in Medicine (DICOM) tag using an ImageJ (version 1.53) Macro function [53]. For completeness, the extracted ATCM profiles for all scanners and experimental setups are available for reference in the Supplementary Materials (Figures S1–S4).

### 2.2. Monte Carlo Simulations

The commercially available, validated Monte Carlo simulation tool ImpactMC (CT imaging GmbH, Erlangen, Germany) was used to simulate and translate the experimental phantom acquisitions into patient-specific CT scans and organ doses [54,55,56,57]. For this, patient models, scanner-specific parameters, and protocol-specific settings are required.

Patient-specific 3D voxel models served as the input for the Monte Carlo dose calculations and were created from retrospectively collected whole-body CT (WBCT) acquisitions. The WBCT images were anonymised, and retrospective use was approved by the institutional ethics committee. A total of 32 patients were selected, ensuring equal numbers of male and female voxel models and, for each sex, an equal proportional distribution of four BMI classes (<18.5: underweight, [18.5–25]: normal weight, [25,26,27,28,29,30]: overweight, >30: obesity).

Scanner-specific input for dose simulations included data on the X-ray spectrum, bowtie filtration, and scanner geometry. The X-ray spectra at tube voltages of 100, 120, and 140 kVp of each scanner were generated using an in-house developed MATLAB (version 9.11.0.1769968 (R2021b)) code (MathWorks, Natick, MA, USA) with an added SPEKTR tool, based on half-value layer measurements using a calibrated pencil beam ionisation chamber (Model 10X6-3CT, Radcal Corporation, Monrovia, CA, USA). Generated spectra for each CT scanner model can be consulted in Supplementary Figure S5. For each scanner, we also measured beam dose profiles free-in-air with the same pencil-beam ionisation chamber while keeping the X-ray tube stationary. The measurements across the axial plane served as input data for the beam-shaping filter thickness in the ImpactMC software (version 1.2.6). Both measurement methodologies are based on and described in detail by Verfaillie et al. [58]. Specifications of the scanner fan angle and distance between the focal point and the isocentre were derived from published manuals or provided by the manufacturer. To calibrate the simulation software, and scale the absorbed doses to a specific scanner, air kerma was measured free-in-air at the isocentre of each at 100, 120, and 140 kVp using the aforementioned ionisation chamber [58]. Scanner-specific actual beam collimation was determined using Gafchromic EBT3 self-developing radiographic film (Ashland Medical, Wilmington, DE, USA).

Based on the ESTI guidelines for lung cancer screening [50,52], 100 kVp was used for CT simulation of underweight voxel models, 120 kVp for the normal and overweight categories, and 140 kVp for the voxel models with obesity. Scanner-specific table increment per rotation was calculated as the beam collimation multiplied by the pitch. The number of rotations depended on the voxel model’s scan range, spanning from the lung apices to the lung base. Variations in scan direction and horizontal and vertical off-centring were simulated by specifying different start z-positions, as well as x- and y-coordinates of the centre of rotation. Tube current files extracted from the voxel models’ original series were considered the ground-truth reference ATCM profile. Changes in ATCM behaviour resulting from variations in scout or scan protocol were modelled in the patient-specific models using the tube current data derived from the RANDO phantom acquisitions. Slice-by-slice comparisons were performed between the phantom tube currents of the reference scan and those of the deviating scans. For the proportions along the scan range where the deviating scan showed higher or lower tube currents than the reference scan, differences were quantified using a normalised root-mean-square error (NRMSE) factor between the two profiles. These NRMSE factors over the proportions were then translated across a voxel model’s scan range, creating patient- and deviation-specific ATCM profiles. Where reference and deviating tube current profiles were identical, the original tube current values of the voxel model were retained unchanged. Adaptation of tube current profiles was automatised using an in-house developed Python (version 3.9.15) code (Python Software Foundation, Wilmington, DE, USA).

The final output of the simulations consisted of individualised 3D dose voxel maps or distributions. These dose distributions consider all relevant interaction processes and dose depositions of 10^10^ photons. The sampling process was continued until a photon left the voxel model or until its energy fell below 10 keV, at which point it was considered absorbed.

### 2.3. Organ-Specific Dose Estimates

The organs of interest in this study were organs completely situated in the scan range, being the lungs, heart, and breasts (for female voxel models only). Further, we also included the liver and thyroid to evaluate doses to organs located outside or at the border of the scanned body region. Volumes of interest (VOIs) were derived from WBCT images of the voxel models by manual and automatic segmentation tools [59]. The VOIs were overlaid as a mask with the 3D dose distributions simulated for all voxel models and all experimental setups, and the organ doses were calculated.

Absolute organ doses are reported in mGy. Differences in doses between the reference scan and a deviation setup are presented as the relative difference, expressed in percentages and calculated with Equation (1):

(1) $$\mathrm{Relative}\;\mathrm{Dose}\;\mathrm{Difference}\;(\%)\;=\;\frac{\left({Dose}_{Dev}-{Dose}_{Ref}\right)}{\left(\frac{{Dose}_{Dev}+{Dose}_{Ref}}{2}\right)}\times 100,$$

where Dose_Dev_ and Dose_Ref_ are the doses to a certain organ in a given voxel model after simulation of, respectively, a deviating setup and the reference setup for each of the four CT scanners.

### 2.4. Statistical Analysis

All statistical analyses and data visualisations were performed using GraphPad Prism software (version 8.0.2) for Windows (GraphPad Software Inc., San Diego, CA, USA). One-Way Repeated Measures Analysis of Variance (ANOVA) was applied to study the effect of variation in the scanogram angle, as well as to examine the differences in doses of each individual organ between ideal positioning and vertical and horizontal off-centring. Dunnett’s correction for multiple comparisons post hoc testing was performed. Organ-specific dose differences resulting from craniocaudal scan direction versus caudocranial direction were compared by means of paired sample *t*-tests. (Dis)agreement of the dose differences obtained after single localiser and dual localiser in different scan scenarios was assessed using Bland–Altman analysis. Hypotheses were tested two-sided, and values of *p* < 0.05 were considered statistically significant.

## 3. Results

A comprehensive overview of the simulated absolute organ doses for each CT scanner model and for the different localizer and scan variations is provided in the online Supplementary Materials. The following sections of the results focus on the relative dose differences between different setups.

### 3.1. Varying Localiser Projection Angle

**Figure 1:**
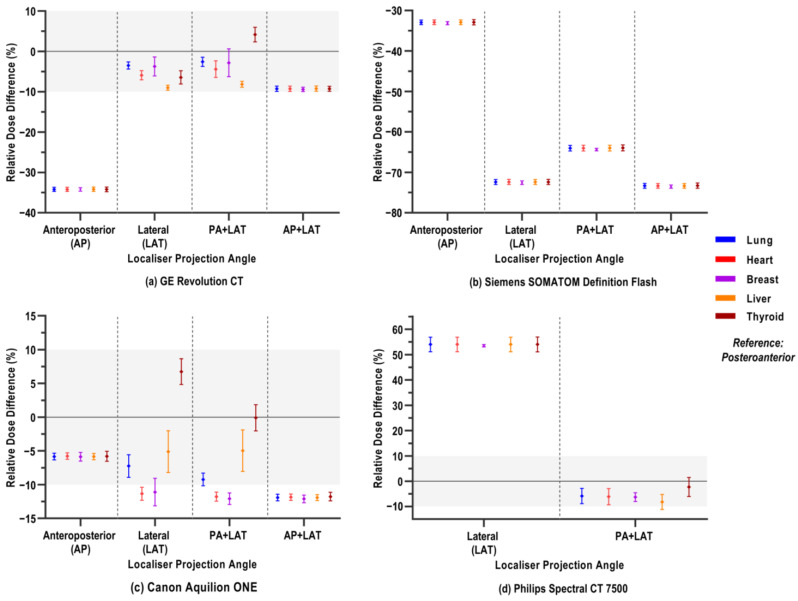
Relative percentage differences in organ doses of a CT chest examination when the scan is preceded by an anteroposterior (AP) or lateral (LAT) localiser or combinations thereof instead of a posteroanterior (PA) projection angle (reference). Mean relative dose difference, averaged over 32 voxel models, is shown with its standard deviation per organ, as depicted in the legend. Different panels show the different CT scanner models: (**a**) GE Revolution CT; (**b**) Siemens SOMATOM Definition Flash; (**c**) Canon Aquilion ONE; (**d**) Philips Spectral CT 7500. Mind that the AP and AP+LAT localiser projection angles were not possible with the Philips model. Note the differing y-axis ranges across graphs. The zone between ±10% relative dose difference is shaded to facilitate visual comparison between varying CT scanner models.

The relative percentage differences in organ doses when the chest CT scan was preceded by localisers from different angles instead of from the PA angle are depicted in Figure 1. Separate panels present results for each CT scanner model. The average organ dose consists of the relative difference values of all 32 voxel models, as every subject was compared against its own reference (PA) dose value, and the distribution of variances was previously checked across all BMI groups. Organ doses and their corresponding relative differences vary between CT scanners. Generally, a PA localiser rendered the highest organ doses, as relative differences in organ doses mostly show decreases upon varying localiser projection angle. There are some cases for thyroid doses (GE, PA+LAT, and Canon LAT, PA+LAT) where a slight increase was observed.

Remarkably, for the Philips scanner, all organ doses were at least 50% higher after a LAT localiser compared to the reference PA (Figure 1d). A LAT localiser combined with a PA localiser for the Philips scanner resulted in a slight decrease of about 5–10% in organ dose. ANOVA multiple comparisons results (Table A1) also show that the absolute organ doses after a LAT and dual PA+LAT localiser are, respectively, significantly higher and lower than for PA. For the Siemens scanner, all organ doses were significantly lower for any other localiser projection angle than PA, with an extreme reduction of up to 73% (Figure 1b). The AP localiser resulted in the greatest dose reduction on the GE scanner (at most 35%) compared to the PA localiser for all organs. For the same scanner, the single LAT and dual PA+LAT resulted in more variable responses across the different organs, but mostly dose reductions (Figure 1a). Only for the breast dose with PA+LAT localisers, no significant differences were observed, while for the same projection angle, the thyroid dose is significantly increased (Table A1). For the Canon scanner, we observed a similar trend of varying dose differences across organs. Here, the thyroid doses are significantly higher for the single LAT localiser or not significantly different from the PA localiser in the case of the PA+LAT dual-localiser setup.

### 3.2. Varying CT Scan Directions

**Figure 2:**
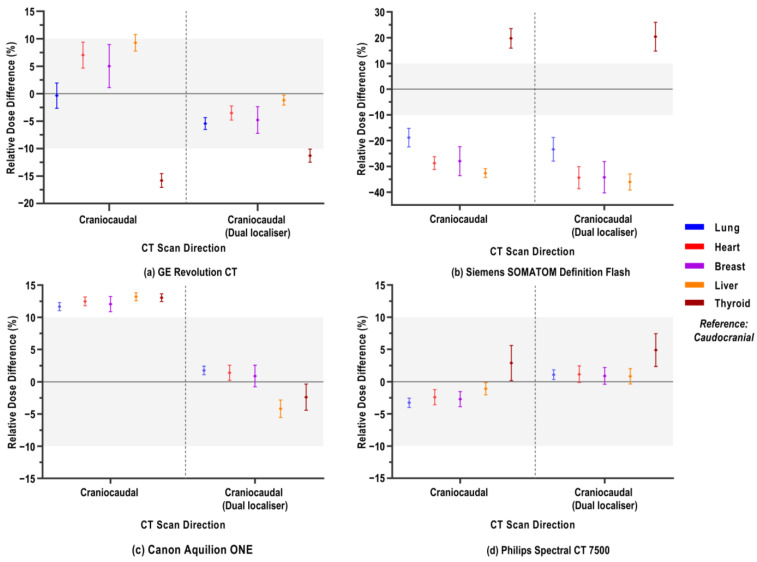
Relative percentage differences in organ doses of a CT chest examination when the CT scan direction is craniocaudal as compared to the reference caudocranial scan direction. The change in scan direction was preceded by either an anteroposterior (AP) or dual (PA+LAT) localiser. Mean relative dose difference, averaged over 32 voxel models, is shown with its standard deviation per organ, as depicted in the legend. Different panels show the different CT scanner models: (**a**) GE Revolution CT; (**b**) Siemens SOMATOM Definition Flash; (**c**) Canon Aquilion ONE; (**d**) Philips Spectral CT 7500. Note the differing y-axis ranges across graphs. The zone between ±10% relative dose difference is shaded to facilitate visual comparison between varying CT scanner models.

Figure 2 shows the relative differences in organ doses when the CT scan direction was changed from caudocranial to craniocaudal. This scenario was either preceded by a single PA localiser or a dual PA+LAT acquisition.

#### 3.2.1. Varying CT Scan Directions After Single Localiser Projection

We refer to the left part of each graph panel, together with Table A2, which presents the paired *t*-test results comparing organ doses for a caudocranial (reference scan) versus a craniocaudal CT scan. Organ doses clearly depend on CT scanner type, but also on the type of organ itself. Overall, the dose differences were limited, typically within an absolute range of 10 to 15% difference. Only for the Siemens scanner, the relative organ dose difference could be substantially larger, showing both pronounced increases and decreases.

For the scanner types of GE, Siemens, and Philips, thyroid doses generally differed from the pattern observed for the other organs. For the Siemens scanner, lung, heart, breast, and liver doses were significantly (up to 32.5%) lower when scanning craniocaudally, whereas the thyroid dose in this setup was considerably higher (19.8% mean increase). This situation is approximately reversed in the case of the GE scanner. Heart, breast, and liver received a higher dose (mean increase of 7.0, 5.0, and 9.3%, respectively), and the thyroid dose was significantly lower (−15.8%) in the case of the craniocaudal scan direction. While differences in doses can be statistically significant, the magnitude of mean dose differences can vary greatly between organs and scanners (Table A2). Also, for the GE scanner, no significant difference was observed in lung dose between both scan directions (−0.4%). Changing scan direction on the Philips scanner type resulted in small dose decreases (lung: −3.3%, heart: −2.4%, breast: −2.7%, liver: −1.1%) and an increase (thyroid: +2.9%). Although small, only the liver doses were shown to be insignificantly different (Table A2). For the Canon scanner, all organs showed an approximately equal percentage of relative dose increase upon variation in the scan direction (Figure 2), which is statistically significant (Table A2).

#### 3.2.2. Varying CT Scan Direction After Dual Localiser Projection

The right half of each graph in Figure 2 shows the relative differences in organ doses when the change in scan direction was preceded by a dual scanogram. Table A3 accordingly indicates the relation between the left and right parts of each graph through Bland–Altman analysis. This analysis assesses how much agreement there is between the outcomes of two methods in terms of bias. In the present study, this relates to the change in doses when a variation in CT scanning protocol is preceded by a dual instead of a single localiser in terms of relative percentage dose difference. A summary of the biases between the two methods is depicted in Table A3, combined for all BMI classes, as the Bland–Altman analysis allows for case-by-case matched comparisons. A positive bias indicates that the organ dose was higher after a craniocaudal CT scan direction preceded by a single PA localiser instead of the same scan direction preceded by a PA+LAT localiser.

Remarkably, for the Siemens scanner, none of the organ doses differed significantly when using dual localiser projection angles compared with the single localiser. This can also be seen in Figure 2b. For the Canon scanner, on the other hand, all evaluated organs received significantly lower doses (mean organ dose differences ranging from 9.9 to 17.4%) when scanning craniocaudally after a dual localiser versus a single localiser (Table A3). For the liver and thyroid doses, there was an additional relative reduction (on average, −4.2 and −2.4%, respectively) observed compared to the caudocranial CT scan direction (Figure 2c). The number of localisers combined with the CT scan direction change did not significantly affect doses to organs outside the scan range on the Philips scanner. However, small but statistically significant increases were observed for lung, heart, and breast doses, as reflected by the negative biases in Table A3. For the GE scanner, organ doses were significantly lower after dual localiser projection. The thyroid dose was on average 4.5% increased after dual instead of single localiser combined with craniocaudal direction (Table A3), but still 11.3% lower compared to the caudocranial scanning (Figure 2).

### 3.3. Vertical Off-Centring

**Figure 3:**
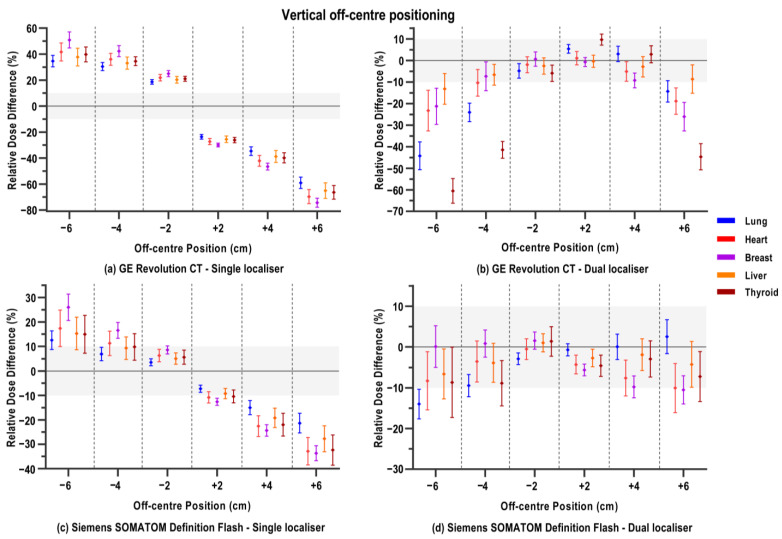

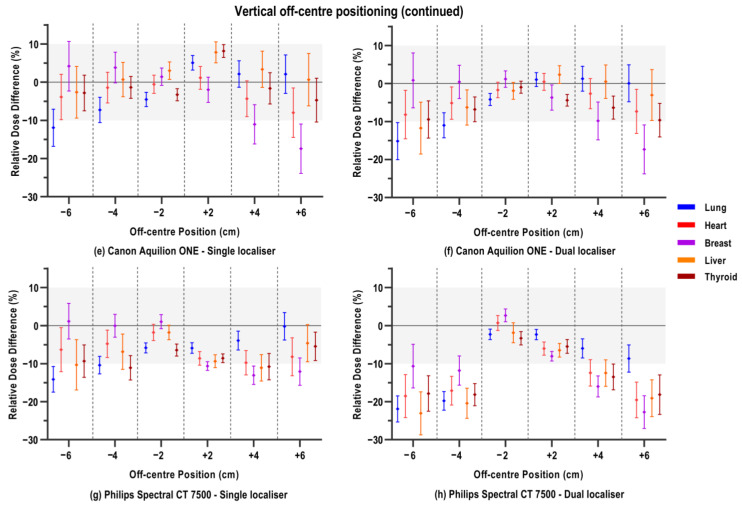
Relative percentage differences in organ doses of a CT chest examination when the participant is positioned too high (+) or low (−) with respect to the scanner isocentre, and where either a single PA or dual PA+LAT localiser is acquired. Mean relative dose difference, averaged over 32 voxel models, is shown with its standard deviation per organ, as depicted in the legend. Different panels show the different CT scanner models: (**a**,**b**) GE Revolution CT; (**c**,**d**) Siemens SOMATOM Definition Flash; (**e**,**f**) Canon Aquilion ONE; (**g**,**h**) Philips Spectral CT 7500. Note the differing y-axis ranges across graphs. The zone between ±10% relative dose difference is shaded to facilitate visual comparison between varying CT scanner models.

Results for off-centre positioning are illustrated in Figure 3 for examinations performed with either a single or dual localiser. Two distinct response patterns were observed among the four scanner models. Firstly, the GE and Siemens scanners showed significant dose increases in all organ doses when the patient was positioned too close to the tube during localiser acquisition (Figure 3a,c). With every downward 2 cm increment, the doses linearly increase due to the magnification of the patient on the localiser image. This linear increase was more pronounced for the GE scanner, where doses across all organs increased between 4.8 and 18.7% per 2 cm stepwise decrease in the table height. These dose increases are significantly different from the absorbed organ doses in the ideal reference position, as can be seen in Table A4. While the dose increases for the Siemens scanner upon mispositioning are smaller with respect to the GE scanner, Table A4 also depicts that they are significantly higher than the organ doses from the reference scan. For Siemens, the dose increases were between at least 3.37% and at most 9.47% per 2 cm downward shift in the table. Both scanners similarly showed a decrease in organ doses when patients were positioned too high with respect to the gantry centre in combination with a single localiser. The minification of patient dimensions reduces the ATCM output, resulting in significant organ dose reductions for both scanner models (Table A4).

Similarly, for both scanners, the pronounced linear dose increases observed with downward off-centre positioning were largely mitigated by using a dual localiser. This can be seen in Figure 3, panels b and d. This is also reflected in the Bland–Altman analysis results in Table A5, where the comparison of a given y-position with either a single or a dual localiser shows that the former results in a much larger relative percentage dose difference compared to the latter. Also, for both scanners, the higher table positions (y+) mostly, but not exclusively, resulted in lower organ doses compared to the reference position. However, the decreases are more moderate here. Generally, for the Siemens scanner, vertical off-centring with the dual localiser resulted in relative dose differences of about −10.0% to maximally −15.0%. The GE scanner, on the other hand, displayed larger dose differences. For example, thyroid doses at y − 6 and y + 6 are, on average, −60.5% and −44.7% below the reference thyroid dose, respectively.

For the Canon and Philips scanners, a second type of ATCM behaviour was observed upon vertical off-centre positioning. When the examination in an off-centre position included a single PA localiser (Figure 3e,g), no pronounced dose increases or decreases were observed for both scanners compared to the GE and Siemens models. On the Canon scanner, average relative dose differences fluctuated between 8.2% (thyroid dose at y + 2 cm) and −8.0% (heart dose at y + 6 cm). Some larger dose reductions were observed for the more extreme off-centre positions, such as −11.9% lung dose at y − 6 cm and −11.0% and −17.4% for the breast dose at respective positions y + 4 cm and y + 6 cm. Table A4 shows no distinct pattern of significant differences in absolute doses for particular organs and/or vertical off-centre positions. While some comparisons with the reference position showed significant differences, the mean differences remained rather low (at most 1 mGy). Doses on the Philips scanner showed decreases in average relative differences compared to the reference scan in almost all off-centre positions. Some negligible and insignificant increases (see also Table A4) were seen in breast doses at the lowered table heights, while generally for all other organs the dose decreases mostly varied between –5.0 and −10.0% at this table height.

Introduction of the additional lateral localiser for the Canon and Philips scanners also had a less pronounced effect when vertical mispositioning occurred, as can also be seen from the Bland–Altman comparisons in Table A5. In cases where there was a difference between the scenario with a single or dual localiser, there even seemed to be an additional tube current and dose reduction in the latter case. This is especially observed in the lowest table positions (y − 4 cm and y − 6 cm) on the Philips scanner model.

### 3.4. Horizontal Off-Centring

**Figure 4:**
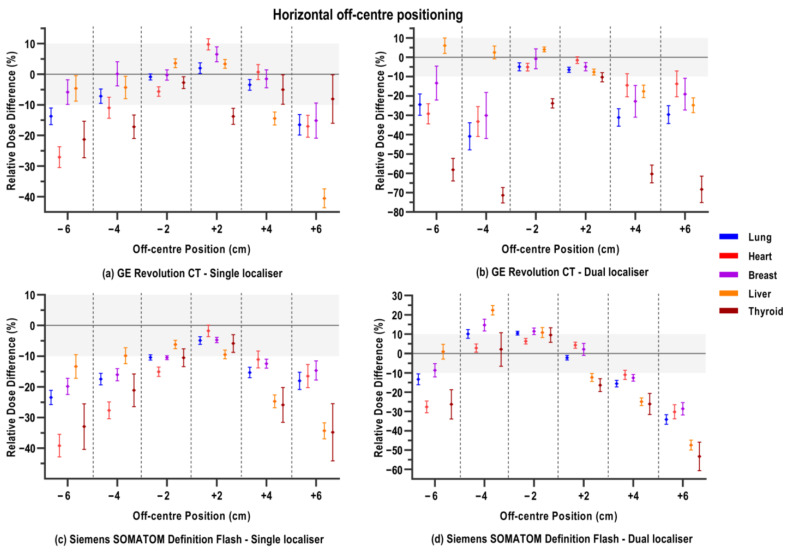

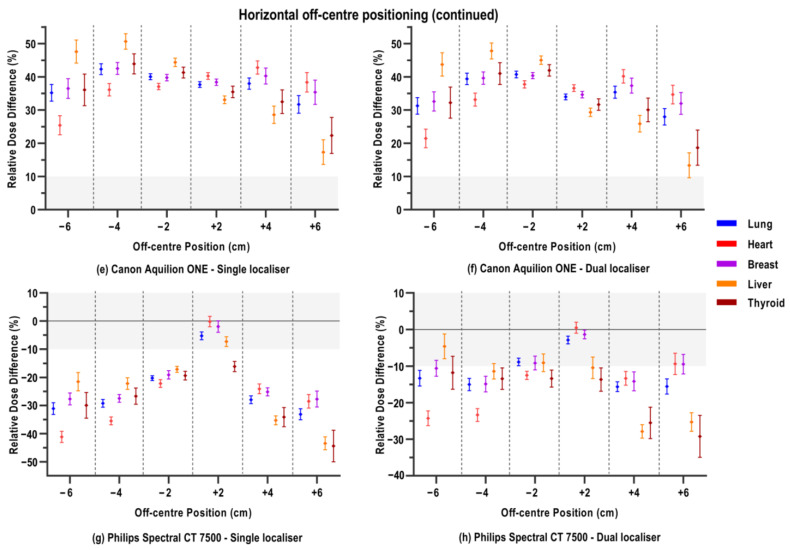
Relative percentage differences in organ doses of a CT chest examination when positioning deviated to the participant’s left-hand (−) or right-hand (+) side with respect to the centreline laser, and where either a single PA or dual PA+LAT localiser was acquired. Mean relative dose difference, averaged over 32 voxel models, is shown with its standard deviation per organ, as depicted in the legend. Different panels show the different CT scanner models: (**a**,**b**) GE Revolution CT; (**c**,**d**) Siemens SOMATOM Definition Flash; (**e**,**f**) Canon Aquilion ONE; (**g**,**h**) Philips Spectral CT 7500. Note the differing y-axis ranges across graphs. The zone between ±10% relative dose difference is shaded to facilitate visual comparison between varying CT scanner models.

Across most scanner models, lateral mispositioning did not show a consistent dose–position relationship. Instead, organ dose changes were variable and, on average, dispersed within a certain organ. However, the most important observation was from the simulations of the Canon CT model. As shown in Figure 4e,f, any horizontal deviation from the optimal position resulted in an apparent complete dysfunction of the ATCM mechanism. Doses for all organs were consistently significantly higher than in the reference position (Table A6). Using a dual instead of a single localiser rendered small but significant dose reductions, except at position x − 2 cm, with about 2–4%. This effect, in the end, is clinically limited, as the average dose differences in lateral mispositioning in combination with a dual scout are still between 13.5% and 47.8% higher than in the reference position.

The remaining three scanners showed a similar trend. Upon lateral mispositioning with a single localiser, average organ doses are lower than in the reference position. Except for the GE scanner (Figure 4a) at positions x − 2, x + 2, and x + 4 cm, all lateral off-centre positions on the three scanner models showed lower average organ doses. As shown in Figure 4 and Table A6, mean organ doses are significantly reduced the more the patient is positioned off-centre; this is especially pronounced for the heart, thyroid, and liver with the Siemens and Philips scanners. What was also observed for the Philips model (Figure 4g) is that off-centre positions beyond 2 cm to the left and mostly to the right resulted in an extreme reduction in the ATCM, with resulting average dose decreases of at least −22.0%. With the Siemens model (Figure 4c), dose decreases are on average more gradual with each 2 cm lateral deviation from the centre.

The additional effect of using a dual instead of a single localiser was less consistent than for the vertical off-centre positioning. For the GE scanner (Figure 4b), thyroid doses are on average increased with respect to the reference scan when off-centre positioning to the left (x−) occurs. Conversely, for the breast doses on this scanner, extreme dose reductions (between −58.1 and −71.3%) were seen from horizontal off-centring beyond 2 cm in either direction, combined with the dual localiser. For the Siemens model (Figure 4, panel d), dose increases were observed upon lateral off-centring to the left (x−); however, in this case, the effect occurred for all organs. Off-centring to the right (x+) mostly rendered equal dose decreases with respect to the reference position, as for the situation with the single localiser. This also translates into the non-significant bias between both setups from the Bland–Altman comparison (Table A7). The dual localiser on the Philips model mostly resulted in a less extreme dose reduction beyond the 2 cm lateral off-centre positions.

## 4. Discussion

With considerably high doses associated with CT compared to other imaging modalities, growing concerns regarding the potential radiation-induced cancer risk have been raised [1,2,3,4]. In response, innovative dose-reduction technologies, such as ATCM systems, have been implemented on almost all CT scanner models [4,6,7,8,20,42,60]. Lung cancer screening represents a particularly relevant application for ATCM techniques. It is of utmost importance to keep the radiation exposure of asymptomatic participants as low as reasonably achievable while they undergo multiple low-dose chest CT scans for either annual screening or follow-up and monitoring of disease progression and nodule growth [36]. As the implementation of a screening programme is bound to increase the workload, deviations from the optimised CT localiser and scan protocol are likely to occur in routine practice. A considerable number of dosimetric studies have highlighted that deviations in CT localiser and scanning protocols can, in fact, alter optimal ATCM functioning and thereby hamper its potential dose-reducing effects. However, current published results stem either from studies with non-anthropomorphic test objects or from investigations limited to a small number of deviating setups on one or two scanner models. In these studies, the primary outcome of interest was mostly a generic dose output, such as CTDI_vol_ or dose-length product [4,10,11,12,15,16,17,18,19,21,22,28,29,30,33,39,45,46]. To address these limitations, we conducted a systematic simulation study of ATCM behaviour under protocol deviations on one CT scanner model from each of four major manufacturers. We specifically characterised potential clinically relevant dose deviations for five organs during chest CT examinations, where variations in the number and angle of localiser projection angles, deviations in CT scanning direction, or off-centre positioning are introduced in practice.

The significant organ dose increases observed in this study underscore the importance of accurate patient/participant positioning. Nevertheless, suboptimal positioning is reported to be highly prevalent in routine practice, especially in the vertical direction [16,29,32,40,43]. In one retrospective study, vertical off-centring beyond 1 cm was reported to occur in 53% of patients, with a maximum posterior shift of 5.4 cm [32]. The results from Deweese et al. also confirm the general tendency of radiographers to set the table height below the isocentre; across 12,464 CT examinations, the average vertical deviation was −1.96 cm (standard deviation 2.48 cm) [43]. The studies focusing on the CTDI_vol_ output reported how these positioning errors affect the function of the ATCM system and how it thereby may diminish its intended dose-saving benefit [39]. In our study, we also observed that only a few centimetres of off-centring, such as the reported 1.96 cm [43], can cause highly significant variations in organ doses. This emphasises that, although known to be extremely prevalent, the effect of suboptimal positioning on (organ) dose metrics should not be underestimated [15].

More recent technological advancements have been marketed to allow for more accurate patient positioning or reduce the impact of miscentring on organ dose variability. Examples include 3D cameras that detect patients’ body contours and adjust the table height accordingly, or positioning compensation systems that adjust ATCM output based on patient dimensions on the localiser image [3,19,21,32,37,43,44]. While these technologies have been proven to have potential, their implementation remains restricted by factors like high cost and limited availability across vendors [3,19,33,39,43,44]. Until then, manual positioning by the radiographer remains standard practice, and proper training and education are paramount, as is further supported by the results of the present study [43].

An effective strategy identified in this study to prevent organ dose increases due to suboptimal positioning is the use of a dual localiser. This approach can be readily implemented into the complete scanning workflow and may be particularly relevant in the case of downward off-centre positioning (Table A5). The additional lateral localiser appears to counteract and even overcompensate for the oversized patient dimensions on the PA localiser projection, thereby limiting excessive ATCM output, which results in significant reductions in organ doses [3,12,16,17,29,35]. However, the true added value of a dual localiser appears to be dependent on the scanner type. The ATCM systems of the Canon and Philips CT scanners appear to have an intrinsic mechanism that compensates for an excessively large or small tube current output when the patient dimensions are, respectively, perceived too large or too small with inadequate table heights. While the dual localiser for these two models still results in significant dose reductions in the actual CT scan, the clinical organ dose differences are more limited than for the GE and Siemens vertical off-centre positioning. As such, the use of two orthogonal localiser projection angles may be considered, depending on the scanner model and its underlying ATCM mechanism, to help ensure that organ doses remain as low as anticipated even in the event of off-centre positioning.

The impact of horizontal off-centring on the ATCM systems has been markedly less investigated than vertical off-centring. Positioning in the horizontal direction is generally facilitated by bilateral symmetry in patients, and as such, lateral off-centre positioning is reported to be much less common [29,32,42,43]. One retrospective study reported that 85% of patients were positioned correctly within 1 cm [29]. Nonetheless, perfect centring is unlikely in all cases because the visually determined centreline is not coincident with the patient’s centre of mass [22]. As such, the standardised setup of the present study allowed for an exhaustive characterisation of important organ dose deviations in multiple hypothetical situations, including horizontal off-centring. Our results indicate that the smaller off-centre deviations (x ± 2 cm), which are more likely to occur in clinical practice, are the only positions to result in organ dose increases (Table A6). In this scenario, the added benefit of the dual scanogram is less pronounced on most CT scanner models (Table A7). In contrast, the Canon model showed important organ dose increases in all horizontal off-centre position setups. As such, while one could argue that horizontal off-centring is less prevalent, it is important to characterise how a specific CT scanner model and its associated ATCM system react to these possible deviations in routine practice. Furthermore, our findings emphasise the importance for radiographers to understand and stay attentive to how mispositioning and choices in CT imaging parameters affect the applied ATCM and potentially compromise its optimal functioning.

The combination of vertical off-centre positioning and a dual localiser resulted in a decrease of almost all organ doses across all scanners. This apparent overcompensation has also been reported with a commercially available positioning compensation system [32]. One study observed an overcompensation in radiation output with a decreasing trend in dose measurements for increasing table heights [32]. While not using a dedicated positioning compensation system, our setup showed a similar overcompensation in the radiation output and organ doses with the use of a dual localiser. However, in this study, the overcompensated, decreased doses were observed in both higher and lower table heights away from the centre. Furthermore, irrespective of whether a single or dual localiser was used, there was an interplay between the misalignment of the patient in the scanner gantry and the bowtie filtration. The purpose of these filters is to spatially shape radiation output emitted from the fan beam in the scanning field of view in order to project maximum X-ray intensity to the thickest region of the patient that attenuates the most, and vice versa [27,28,32,34,38,39,41]. However, this assumes that the patient is accurately located in the centre of the beam. The combined effect of off-centre positioning and the bowie shape filter can result in additional dose increases and decreases for particular organs [38]. Anteriorly positioned organs, such as the thyroid and breast, which are normally projected in the thinner and less attenuating parts of the filter during scan rotation, are exposed to a higher intensity central part of the beam when patients are positioned too low, for example [9,32,35,38]. Shifting bilateral organs towards or away from the centre can result in additional complex effects on the dose distributions, with one side of the organ experiencing notably higher exposure [32]. On the other hand, for larger organs, the effect could be averaged out as compared to asymmetric organs, such as the liver [38]. While our study setup with simulated organ-specific dose distributions provided more insights into this behaviour than a summarising CTDI_vol_ value, we did not quantify the specific contribution of the bowtie filtration to the observed dose results.

In addition, several retrospective studies have shown that the substantial variability in patient dose is not only caused by suboptimal positioning but, importantly, also by other different parameters in the imaging protocol, such as localiser angles and CT scan direction [6,28,35,42]. Harri et al. reported that the dose-length product between two examinations of the same patient on the same CT scanner can vary up to 238% solely because of the actions and choices of the radiographer [22]. That study also noted that protocol changes are sometimes implemented by the radiographer without a clear rationale. For example, localiser projection angles were changed from AP to PA by CT operators who believed that the latter would expose the patient to less radiation dose [22]. However, the results in present study demonstrate that such seemingly minor, unsubstantiated deviations from the optimised protocol can have significant influences on the absorbed organ doses from the subsequent helical CT scan. However, the literature reviews indicate that comprehensive guidance on the optimal number or projection angle needed to optimise ATCM functioning remains limited [3,4]. Moreover, results from experimental setups also present various important considerations. Due to the divergence of the X-ray beam, structures located closer to the tube during localiser acquisitions appear enlarged. As such, several studies found that the dose of a diagnostic scan was markedly higher after a PA localiser compared to the AP localiser, as the magnified view of particularly the vertebrae and ribs led to higher tube current output [4,12,16,27,28]. The organ dose simulations after varying localiser angles in this study do confirm these findings for the GE, Siemens, and Canon models, in which we could make direct comparisons between organ doses of examinations preceded by either a PA or AP localiser. In all cases, the PA projection led to the highest doses. Only for the Philips scanner model, we observed that doses were not highest after the PA localiser, but after the lateral one. However, the use of a single lateral localiser could be discouraged as it is reported to have notably reduced image quality [3,30]. On the other hand, it has also been argued that there should actually be an option to use a PA projection to reduce radiation dose to the breasts and other anteriorly located radiosensitive organs [3,30,31]. Ultimately, several arguments can be advanced in support of the different localiser projection angles. However, it appears that these results are highly dependent on the scanner type, as is confirmed by the simulated dose results in the present study.

Our results suggest that the application of two orthogonal localisers prior to the CT examination is not detrimental to optimal ATCM functioning. In fact, it can be beneficial to compensate or prevent dose increases from suboptimal positioning, as previously discussed. This is consistent with prior reports showing a significant reduction in CTDI_vol_ in all patients for both chest and abdomen examinations when two projections were used compared to a single PA localiser [3,17,30]. These results contrast with publications and recommendations advocating the use of a single localiser radiograph to minimise overall dose to the patient or participant [15,17]. However, the dose increase from an additional localiser acquisition is small relative to the potential excessive dose increases resulting from patient magnification and associated higher tube current selection [3,10]. Therefore, the minor dose increase in an additional localiser may be worth considering in the context of lung cancer screening [10]. Overall, given the marked variability across scanner models, there is no consensus on which projection angle is optimal for all scenarios. As such, the localiser protocol should be subject to an equally thorough and scanner-specific optimisation process as the helical CT scanning protocol itself [16].

Lastly, we simulated the effect on the organ doses after varying the scan direction during the helical CT scan. Overall, this deviation is relatively less studied compared to variation in localiser projection angle [24,26,45,46]. Among the available evidence, one study presented lung and thyroid doses from Monte Carlo simulations on the same Siemens Definition Flash scanner model as used in the present study [26]. Franck et al. similarly observed an increase in the thyroid dose when scanning in the craniocaudal direction (from 5.7 mGy to 10.2 mGy). Here, the lung also received less dose, but the dose decrease was relatively smaller than in our results (from 10.7 mGy to 9.5 mGy) [26]. They also performed dose simulations with the varying scan direction combined with a dual localiser projection. However, they used an AP+LAT localizer, in contrast to the PA+LAT setup in the present study. As such, direct comparison of these results is difficult. Yet, in the results of the present study, we see that the added LAT scanogram has no clinically significant effect on the lung and thyroid doses of the Siemens scanner when scanning in the craniocaudal direction compared to the single AP localiser. However, for Franck et al., the doses were observed to be half as big after the AP+LAT localiser and craniocaudal scan direction compared to the single AP localiser and craniocaudal scanning [26]. Generally, when looking at the variation in scan direction in the present study, there can mostly be a difference observed between the dose results of Siemens scanners on the one hand and GE, Canon, and Philips scanners on the other hand. For the Siemens model, craniocaudal scan direction was consistently associated with a significant increase in the thyroid dose. A potential explanation relates to the underlying mechanism of the ATCM systems. Siemens ATCM system (CARE Dose 4D) has an additional real-time, online modulation of the X-ray tube current output, whereas for the systems of GE (SmartmA), Canon (SureExposure 3D), and Philips (DoseRight), the ATCM is predefined and totally based on the attenuation data of the localiser image [6,7,11,24,26]. Nonetheless, it has been observed that Siemens’ CARE Dose 4D depends on where the scan starts; the first 180° of rotation operates without real-time modulation, and it is not uncommon that mA values are higher when the acquisition begins in the neck region [24].

This study demonstrates that different CT scanner models are equipped with varying ATCM techniques that also differ substantially in their response to deviating localiser and scanning setups. However, there are numerous other factors that vary between vendors and scanners. These variations in, for example, the focus to isocentre distance and bowtie filtration will also alter the extent of the effect of a particular deviation [6,11,27,39,42]. Some studies reported differences in ATCM behaviour, even between scanners from the same manufacturer [11,27]. Consequently, the present results cannot be directly generalised to all existing scanner models across or even within vendors. These findings nonetheless reinforce the need for clinical scan protocols to be developed and optimised in close collaboration with medical physics experts, according to the scanner-specific ATCM system, and by gaining insights into its limitations and behaviour in deviation setups [6,9,16,23]. In addition, manufacturers could contribute to this radiation dose optimisation by providing clearer, scanner-specific recommendations or guidelines for acquisition or localiser parameters [11,17,22]. Such guidance would also facilitate targeted radiographer training and improve understanding of how protocol choices and positioning deviations affect ATCM performance of a given system.

Although Monte Carlo simulations enable the estimation of organ- and patient-specific doses beyond averaged scanner output metrics such as CTDI_vol_, this methodology remains subject to several sources of uncertainties. Firstly, there is an inherent organ dose uncertainty related to the input parameters used in the Monte Carlo simulation technique [61]. Scanner-specific characteristics, such as the X-ray spectrum or bowtie geometry, are proprietary data from the manufacturer and also had to be modelled based on measurements. Moreover, the dynamic alteration of the ATCM during a CT examination remains challenging to accurately model for a specific scanner model as input for the simulation software [62]. In addition, the proposed method to translate tube current profiles from a phantom setup to the voxel model could be the subject of in-depth, validating research. A combination of the abovementioned factors impedes generalisation of the absorbed organ dose estimates in this study as an absolute ground-truth dose value. However, the primary aim of this study was to systematically and extensively characterise possible changes in ATCM behaviour upon localiser and scan deviations and translate this to consequential dose implications. Therefore, to eliminate the influence of the inherent uncertainties on the absorbed dose values, this study mostly focused on and provided results in terms of relative differences between deviating setups. Another considerable drawback in this study was the use of one anthropomorphic phantom with a relatively lean habitus. Importantly, there were also no phantom breast attachments to represent the female voxel models during experimental CT scanning. Additional future research with phantoms representing female patients or patients with larger dimensions could determine whether ATCM behaviour changes differently for these other patient variabilities.

Our results suggest that the use of a dual localiser in most deviations ensures that the ATCM systems perform at least equally well as in the case of a single localiser. Depending on the CT scanner model, an additional localiser projection even mitigates significant dose increases that were present with only a single localiser, such as for the simulations of inaccurate off-centre positioning. While the application of a dual localiser as such might be an important consideration, the authors do realise that we did not specifically simulate and calculate the radiation output from the localiser acquisitions themselves. Future work will evaluate whether the incremental organ doses from the additional scout are consistently negligible and could support the development of recommendations for localiser protocols.

Future research will also evaluate the findings from the present study from an image quality perspective. Given the critical trade-off between radiation dose and image quality, the observed relative dose decreases in some experimental deviating setups are predicted to be detrimental to the image quality. In particular, an increase in image noise may impair low contrast resolution and, consequently, diagnostic performance [5,7,36,46]. Accordingly, future studies will extend our in-depth evaluation of ATCM behaviour by examining resulting image quality parameters across various CT scanner models using standardised reconstruction parameters. As such, the dose results of the present study without image-quality assessment warrant cautious clinical interpretation. The exact explanations for the differences in dose variations between CT scanners and across different setups are difficult to establish. Mechanisms of ATCM systems remain proprietary and are fundamentally designed to maintain a predefined level of image quality rather than to directly control dose [7,16,63,64]. Therefore, their precise operational mechanisms are only partially transparent. Although all systems are based on the general principle of image quality optimisation, variations in their implementation may contribute to the differing dose trends observed in this study [7,16]. However, considering the primary focus on dose, only limited mechanistic interpretation is possible in the present study. The proposed complementary investigation of the image quality might therefore help unravel the mechanisms underlying the dose-related trends. In an additional small-scale study, we will investigate implications for organ-specific doses when the organs of interest scanned during helical CT examination are not entirely included in the localiser acquisition. Prior studies have reported that limited localiser data can cause erratic compensation of the ATCM system, resulting in excessively high or low tube currents [7,16]. Characterisation of this behaviour for specific CT models could be important considering the presence of sensitive organs, such as the thyroid, located at the edge of the scan range in chest examinations. Also, regarding the dose of organs outside of the scan range, standardised comparison between the dose due to z-overscanning on different multirow detectors of varying vendors is of major interest [65,66,67]. Future simulation-based research regarding z-overscanning and its potential interaction with, for example, off-centre positioning could complement the results presented in this manuscript.

## 5. Conclusions

Despite the recognised high frequency of patient mispositioning and CT protocol variations in routine practice, the results of this simulation study demonstrate that their implications on organ-specific doses are significant and should not be overlooked, risking underestimation of anticipated CT imaging radiation doses. Moreover, marked variability in ATCM behaviour was observed across scanner models from four different major vendors. Consequently, the development and optimisation of CT scanning and localiser protocols should be supported by medical physics expertise, ensuring a thorough scanner-specific understanding of ATCM systems. This, in turn, can contribute to radiographer training and promote consistent, adequate operation of a specific scanner in practice.

## Data Availability

The original contributions presented in this study are included in the article. Further inquiries can be directed to the corresponding author.

## Supplementary Materials

The following supporting information can be downloaded at: https://www.mdpi.com/article/10.3390/jimaging12030123/s1, Figure S1: Automatic tube current modulation (ATCM) profiles derived from the experimental scanning setups with the RANDO phantom on the GE Revolution CT scanner; Figure S2: Automatic tube current modulation (ATCM) profiles derived from the experimental scanning setups with the RANDO phantom on the Siemens SOMATOM Definition Flash scanner; Figure S3: Automatic tube current modulation (ATCM) profiles derived from the experimental scanning setups with the RANDO phantom on the Canon Aquilion ONE scanner; Figure S4: Automatic tube current modulation (ATCM) profiles derived from the experimental scanning setups with the RANDO phantom on the Philips Spectral CT 7500 scanner; Figure S5: Generated X-ray spectra used as scanner-specific input for Monte Carlo dose simulations; Table S1: Overview of the absolute organ doses from Monte Carlo simulations where the CT scan is preceded by a localiser from varying angles; Table S2: Overview of the absolute organ doses from Monte Carlo simulations where the CT scan direction is changed to craniocaudal, preceded by either a single PA or dual PA+LAT localizer; Table S3: Overview of the absolute organ doses from Monte Carlo simulations where the voxel models were positioned off-centre in the vertical direction, preceded by a either a single PA or dual PA+LAT localiser; Table S4: Overview of the absolute organ doses from Monte Carlo simulations where the voxel models were positioned off-centre in the horizontal/lateral direction, preceded by either a single PA or dual PA+LAT localiser.

## Abbreviations

The following abbreviations are used in this manuscript:

CT	Computed tomography
AEC	Automatic exposure control
ATCM	Automatic tube current modulation
CTDI_vol_	Volume computed tomography dose index
PA	Posteroanterior
AP	Anteroposterior
LAT	Lateral
ESTI	European Society of Thoracic Imaging
DICOM	Digital Imaging and Communications in Medicine
WBCT	Whole-body CT
NRMSE	Normalised root-mean-square error
VOI	Volume of interest
ANOVA	Analysis of variance

## Appendix A

**Table A1:** Results of the one-way analysis of variance (ANOVA) of the absolute organ doses. Per the CT scanner brand depicted are the multiple comparisons for the organ doses after posteroanterior (PA) localiser projection angle compared to the doses after anteroposterior (AP) or lateral (LAT) localiser or combinations thereof. Values are the mean difference in the comparisons with the standard error of the difference between brackets. *p*-values are adjusted according to Dunnett’s correction. Significance codes: ****: *p* < 0.0001; ***: *p* < 0.001; **: *p* < 0.01; *: *p* < 0.05; ns: not significant.

Varying Localiser Angle|ANOVA Results						
Scanner	Comparison	Organ				
		Lung	Heart	Breast	Liver	Thyroid
GE Revolution CT	PA vs. AP	1.77 (0.19) ****	1.77 (0.18) ****	2.10 (0.32) ****	1.40 (0.15) ****	1.97 (0.19) ****
	PA vs. LAT	0.21 (0.025) ****	0.34 (0.036) ****	0.26 (0.059) **	0.41 (0.043) ****	0.41 (0.042) ****
	PA vs. PALAT	0.14 (0.018) ****	0.22 (0.023) ****	0.15 (0.071) ns	0.36 (0.037) ****	−0.29 (0.040) ****
	PA vs. APLAT	0.54 (0.060) ****	0.54 (0.057) ****	0.65 (0.096) ****	0.43 (0.047) ****	0.59 (0.056) ****
Siemens SOMATOM Definition Flash	PA vs. AP	1.06 (0.13) ****	1.04 (0.13) ****	1.40 (0.25) ***	0.78 (0.097) ****	1.17 (0.13) ****
	PA vs. LAT	1.82 (0.23) ****	1.79 (0.22) ****	2.40 (0.42) ***	1.35 (0.17) ****	2.02 (0.23) ****
	PA vs. PALAT	2.01 (0.26) ****	1.98 (0.24) ****	2.65 (0.47) ***	1.49 (0.18) ****	2.23 (0.25) ****
	PA vs. APLAT	1.99 (0.25) ****	1.96 (0.24) ****	2.63 (0.47) ***	1.47 (0.18) ****	2.20 (0.25) ****
Canon Aquilion ONE	PA vs. AP	0.20 (0.039) ****	0.20 (0.037) ****	0.33 (0.046) ****	0.17 (0.030) ****	0.25 (0.035) ****
	PA vs. LAT	0.63 (0.064) ****	0.64 (0.060) ****	0.67 (0.095) ****	0.50 (0.049) ****	−0.40 (0.043) ****
	PA vs. PALAT	0.50 (0.054) ****	0.63 (0.059) ****	0.67 (0.096) ****	0.23 (0.036) ****	0.0076 (0.020) ns
	PA vs. APLAT	0.40 (0.048) ****	0.60 (0.057) ****	0.61 (0.094) ****	0.24 (0.036) ****	0.65 (0.060) ****
Philips Spectral CT 7500	PA vs. LAT	−5.73(0.59) ****	−5.60 (0.55) ****	−6.65 (1.03) ****	−4.60 (0.49) ****	−6.11 (0.57) ****
	PA vs. PALAT	0.44 (0.061) ****	0.43 (0.052) ****	0.55 (0.093) ****	0.48 (0.060) ****	0.17 (0.067) *

**Table A2:** Results of the paired *t*-test of the absolute organ doses. Per the CT scanner brand depicted are the paired comparisons for the organ doses after a CT scan in the caudocranial direction compared to the craniocaudal direction, both preceded by a posteroanterior (PA) localiser projection angle. Values are the mean difference with the standard error of the mean difference between brackets. Significance codes: ****: *p* < 0.0001; ***: *p* < 0.001; **: *p* < 0.01; ns (not significant): *p* ≥ 0.05.

Varying CT Scan Direction|Paired *t*-Test Results						
Scanner	Comparison	Organ				
		Lung	Heart	Breast	Liver	Thyroid
GE Revolution CT	Caudocranial vs. Craniocaudal	0.029 (0.026) ns	−0.41 (0.045) ****	−0.37 (0.11) **	−0.45 (0.048) ****	0.97 (0.089) ****
Siemens SOMATOM Definition Flash	Caudocranial vs. Craniocaudal	0.66 (0.092) ****	0.91 (0.11) ****	1.22 (0.24) ***	0.77 (0.096) ****	−0.87 (0.097) ****
Canon Aquilion ONE	Caudocranial vs. Craniocaudal	−0.64 (0.092) ****	−0.71 (0.091) ****	−0.66 (0.17) **	−0.57 (0.082) ****	−0.74 (0.10) ****
Philips Spectral CT 7500	Caudocranial vs. Craniocaudal	0.21 (0.046) ****	0.15 (0.043) **	0.24 (0.043) ****	0.024 (0.034) ns	−0.30 (0.078) ***

**Table A3:** Results of the Bland–Altman comparison. Per scanner and organ, the bias indicates how much (dis)agreement in organ doses there is when a deviating scan direction (craniocaudal CT scanning) is preceded by either a single PA or dual PA+LAT localiser (single vs. dual). Values are the mean difference (bias) in percentage with the standard error of the bias between brackets. Significance codes: ****: *p* < 0.0001; ***: *p* < 0.001; **: *p* < 0.01; ns (not significant): *p* ≥ 0.05.

Varying CT Scan Direction|Bland–Altman Test Results						
Scanner	Comparison	Organ				
		Lung	Heart	Breast	Liver	Thyroid
GE Revolution CT	Craniocaudal: single vs. dual	5.09 (1.61) **	10.52 (1.64) ****	10.42 (2.89) ***	10.53 (1.16) ****	−4.46 (1.60) ns
Siemens SOMATOM Definition Flash	Craniocaudal: single vs. dual	5.18 (6.05) ns	6.34 (6.27) ns	6.97 (5.51) ns	3.98 (5.58) ns	−0.62 (5.63) ns
Canon Aquilion ONE	Craniocaudal: single vs. dual	9.93 (1.02) ****	11.11 (1.61) ****	10.91 (1.48) ****	17.40 (1.66) ****	15.48 (2.08) ****
Philips Spectral CT 7500	Craniocaudal: single vs. dual	−4.36 (0.63) ****	−3.57 (0.82) **	−3.61 (0.80) **	−1.93 (0.58) ns	−2.01 (1.61) ns

**Table A4:** Results of the one-way analysis of variance (ANOVA) of the absolute organ doses. The multiple comparisons for the organ doses after off-centre positioning in the vertical (y) direction are depicted per the CT scanner brand. Values are the mean difference in the comparisons with the standard error of the difference between brackets. *p*-values are adjusted according to Dunnett’s correction. Significance codes: ****: *p* < 0.0001; ***: *p* < 0.001; **: *p* < 0.01; *: *p* < 0.05; ns: not significant.

Vertical Off-Centring|ANOVA Results						
Scanner	Comparison	Organ				
		Lung	Heart	Breast	Liver	Thyroid
GE Revolution CT	Ref vs. y − 6	−2.63 (0.33) ****	−3.39 (0.45) ****	−5.11 (0.89) ***	−2.35 (0.32) ****	−3.34 (0.35) ****
	Ref vs. y − 4	−2.25 (0.27) ****	−2.79 (0.35) *****	−4.00 (0.68) ***	−1.98 (0.26) ****	−2.83 (0.29) ****
	Ref vs. y − 2	−1.27 (0.15) ****	−1.53 (0.18) ****	−2.08 (0.34) ***	−1.11 (0.14) ****	−1.58 (0.16) ****
	Ref vs. y + 2	1.29 (0.14) ****	1.47 (0.16) ****	1.87 (0.29) ****	1.09 (0.13) ****	1.55 (0.15) ****
	Ref vs. y + 4	1.80 (0.20) ****	2.13 (0.23) ****	2.71 (0.42) ****	1.56 (0.18) ****	2.21 (0.21) ****
	Ref vs. y + 6	2.76 (0.31) ****	3.15 (0.34) ****	3.89 (0.59) ****	2.34 (0.26) ****	3.33 (0.32) ****
Siemens SOMATOM Definition Flash	Ref vs. y − 6	−0.55 (0.093) ****	−0.82 (0.16) ****	−1.56 (0.33) **	−0.54 (0.11) ***	−0.67 (0.11) ****
	Ref vs. y − 4	−0.30 (0.053) ****	−0.50 (0.095) ****	−0.92 (0.18) ***	−0.32 (0.064) ***	−0.42 (0.071) ****
	Ref vs. y − 2	−0.14 (0.026) ****	−0.26 (0.046) ****	−0.44 (0.083) ***	−0.16 (0.031) ****	−0.23 (0.037) ****
	Ref vs. y + 2	0.26 (0.036) ****	0.38 (0.052) ****	0.57 (0.10) ***	0.25 (0.037) ****	0.39 (0.047) ****
	Ref vs. y + 4	0.52 (0.073) ****	0.74 (0.10) ****	1.06 (0.19) ***	0.49 (0.071) ****	0.79 (0.090) ****
	Ref vs. y + 6	0.72 (0.10) ****	1.025 (0.13) ****	1.41 (0.26) ***	0.68 (0.096) ****	1.11 (0.12) ****
Canon Aquilion ONE	Ref vs. y − 6	0.60 (0.069) ****	0.14 (0.074) ns	−0.38 (0.16) ns	0.066 (0.067) ns	0.17 (0.058) *
	Ref vs. y − 4	0.38 (0.048) ****	0.040 (0.053) ns	−0.30 (0.10) *	−0.058 (0.049) ns	0.084 (0.037) ns
	Ref vs. y − 2	0.24 (0.029) ****	0.023 (0.029) ns	−0.11 (0.051) ns	−0.14 (0.031) ***	0.19 (0.024) ****
	Ref vs. y + 2	−0.28 (0.030) ****	−0.041 (0.036) ns	0.13 (0.064) ns	−0.34 (0.035) ****	−0.51 (0.052) ****
	Ref vs. y + 4	−0.11 (0.041) ns	0.27 (0.072) **	0.67 (0.14) **	−0.13 (0.046) *	0.062 (0.044) ns
	Ref vs. y + 6	−0.11 (0.061) ns	0.47 (0.10) ***	1.00 (0.20) ***	−0.012 (0.068) ns	0.21 (0.061) **
Philips Spectral CT 7500	Ref vs. y − 6	0.93 (0.11) ****	0.32 (0.10) *	−0.19 (0.14) ns	0.46 (0.073) ****	0.68 (0.12) ****
	Ref vs. y − 4	0.69 (0.085) ****	0.25 (0.069) **	−0.044 (0.076) ns	0.29 (0.056) ****	0.82 (0.11) ****
	Ref vs. y − 2	0.39 (0.061) ****	0.058 (0.050) ns	−0.10 (0.057) ns	0.053 (0.040) ns	0.45 (0.076) ****
	Ref vs. y + 2	0.42 (0.065) ****	0.61 (0.082) ****	0.89 (0.13) ****	0.53 (0.076) ****	0.62 (0.078) ****
	Ref vs. y + 4	0.27 (0.060) ***	0.69 (0.097)1 ****	1.09 (0.18) ***	0.64 (0.097) ****	0.77 (0.092) ****
	Ref vs. y + 6	−0.014 (0.062) ns	0.58 (0.11) ****	0.99 (0.17) ***	0.27 (0.083) *	0.35 (0.064) ****

**Table A5:** Results of the Bland–Altman comparison. Per scanner and organ, the bias indicates how much (dis)agreement in organ doses there is when vertical off-centre positioning is preceded by either a single PA or dual PA+LAT localiser (single vs. dual). Values are the mean difference (bias) in percentage with the standard error of the bias between brackets. A positive bias means that the organ dose was bigger for the single PA localiser compared to the dual PA+LAT localiser. Significance codes: ****: *p* < 0.0001; ***: *p* < 0.001; **: *p* < 0.01; *: *p* < 0.05; ns (not significant): *p* ≥ 0.05.

Vertical Off-Centring|Bland–Altman Results						
Scanner	Comparison	Organ				
		Lung	Heart	Breast	Liver	Thyroid
GE Revolution CT	y − 6: single vs. dual	78.97 (5.04) ****	65.12 (6.81) ****	72.34 (9.32) ****	51.1 (4.95) ****	100.2 (1.70) ****
	y − 4: single vs. dual	54.63 (3.15) ****	46.54 (4.51) ****	49.81 (7.20) ****	39.92 (3.41) ****	76.03 (1.43) ****
	y − 2: single vs. dual	23.47 (3.31) ****	23.87 (3.26) ****	24.35 (2.82) ****	22.87 (3.42) ****	26.91 (6.26) ****
	y + 2: single vs. dual	−29.03 (1.44) ****	−28.40 (2.47) ****	−29.16 (1.42) ****	−25.26 (2.01) ****	−35.80 (2.11) ****
	y + 4: single vs. dual	−37.73 (2.09) ****	−37.05 (2.05) ****	−37.16 (1.31) ****	−35.98 (2.95) ****	−42.69 (1.71) ****
	y + 6: single vs. dual	−44.72 (4.19) ****	−50.88 (3.63) ****	−48.04 (5.96) ****	−56.6 (3.64) ****	−21.63 (2.07) ****
Siemens SOMATOM Definition Flash	y − 6: single vs. dual	26.66 (1.11) ****	25.95 (1.48) ****	26.12 (1.79) ****	22.13 (1.76) ****	23.62 (1.89) ****
	y − 4: single vs. dual	16.48 (0.98) ****	15.03 (1.22) ****	15.77 (1.73) ****	13.37 (0.72) ****	18.69 (0.85) ****
	y − 2: single vs. dual	6.47 (0.63) ****	6.88 (1.06) ****	6.99 (1.69) ****	4.11 (1.04) ****	4.24 (1.47) **
	y + 2: single vs. dual	−6.58 (0.63) ****	−6.58 (0.52) ****	−6.95 (0.45) ****	−6.60 (0.44) ****	−5.80 (0.57) ****
	y + 4: single vs. dual	−15.08 (1.61) ****	−15.01 (1.52) ****	−14.47 (1.06) ****	−17.39 (1.90) ****	−18.96 (1.01) ****
	y + 6: single vs. dual	−23.88 (0.63) ****	−22.77 (0.94) ****	−22.93 (1.01) ****	−23.57 (0.66) ****	−24.97 (0.96) ****
Canon Aquilion ONE	y − 6: single vs. dual	3.22 (0.86) ***	4.30 (1.73) *	3.36 (1.96) ns	9.11 (1.18) ****	6.63 (1.16) ****
	y − 4: single vs. dual	3.71 (0.55) ****	3.72 (1.16) **	3.40 (1.06) **	6.98 (0.85) ****	5.44 (1.03) ****
	y − 2: single vs. dual	−0.31 (0.90) ns	1.15 (1.39) ns	0.25 (1.65) ns	4.92 (0.85) ****	−2.32 (1.16) ns
	y + 2: single vs. dual	4.03 (1.23) **	0.69 (1.55) ns	1.69 (2.55) ns	5.47 (1.56) ***	12.58 (1.03) ****
	y + 4: single vs. dual	0.88 (1.14) ns	−1.68 (1.48) ns	−1.20 (2.11) ns	2.88 (1.32) *	4.73 (1.98) *
	y + 6: single vs. dual	2.05 (1.14) ns	−0.67 (1.56) ns	−0.094 (2.20) ns	3.69 (1.29) **	4.93 (2.21) *
Philips Spectral CT 7500	y − 6: single vs. dual	7.79 (1.53) ****	12.2 (1.65) ****	3.36 (1.96) ns	12.75 (1.58) ****	8.52 (3.01) **
	y − 4: single vs. dual	9.39 (0.99) ****	12.34 (1.23) ****	3.40 (1.06) **	13.56 (1.30) ****	7.06 (1.76) ****
	y − 2: single vs. dual	−3.55 (0.71) **	−2.50 (1.12) ns	0.25 (1.65) ns	0.058 (1.38) ns	−3.10 (2.16) ns
	y + 2: single vs. dual	−3.55 (0.58) ****	−2.58 (0.56) **	1.70 (2.55) ns	−2.89 (0.75) *	−3.10 (1.63) ns
	y + 4: single vs. dual	2.06 (0.91) *	2.69 (1.65) ns	−1.20 (2.11) ns	1.38 (0.90) ns	2.68 (1.96) ns
	y + 6: single vs. dual	8.45 (1.30) ****	11.34 (1.77) ****	−0.094 (2.20) ns	14.48 (1.26) ****	12.69 (3.15) ****

**Table A6:** Results of the one-way analysis of variance (ANOVA) of the absolute organ doses. The multiple comparisons for the organ doses after non-ideal positioning in the horizontal (x) direction are depicted per the CT scanner brand. Values are the mean difference in the comparisons with the standard error of the difference between brackets. *p*-values are adjusted according to Dunnett’s correction. Significance codes: ****: *p* < 0.0001; ***: *p* < 0.001; **: *p* < 0.01; *: *p* < 0.05; ns: not significant.

Horizontal Off-Centring|ANOVA Results						
Scanner	Comparison	Organ				
		Lung	Heart	Breast	Liver	Thyroid
GE Revolution CT	Ref vs. x − 6	0.77 (0.086) ****	1.41 (0.14) ****	0.36 (0.098) *	0.17 (0.033) ***	1.32 (0.16) ****
	Ref vs. x − 4	0.42 (0.050) ****	0.65 (0.078) ****	−0.0034 (0.083) ns	0.15 (0.026) ****	1.08 (0.13) ****
	Ref vs. x − 2	0.056 (0.015) **	0.34 (0.042) ****	0.029 (0.031) ns	−0.17 (0.021) ****	0.19 (0.040) ***
	Ref vs. x + 2	−0.13 (0.029) ***	−0.62 (0.069) ****	−0.51 (0.093) ***	−0.16 (0.021) ****	0.84 (0.072) ****
	Ref vs. x + 4	0.20 (0.030) ****	−0.056 (0.033) ns	0.087 (0.060) ns	0.64 (0.065) ****	0.29 (0.056) ****
	Ref vs. x + 6	0.92 (0.11) ****	0.91 (0.094) ****	0.99 (0.19) ***	1.60 (0.17) ****	0.47 (0.096) ***
Siemens SOMATOM Definition Flash	Ref vs. x − 6	0.79 (0.11) ****	1.21 (0.14) ****	0.92 (0.18) ***	0.33 (0.043) ****	1.19 (0.15) ****
	Ref vs. x − 4	0.60 (0.078) ****	0.89 (0.11) ****	0.74 (0.14) ***	0.25 (0.033) ****	0.81 (0.11) ****
	Ref vs. x − 2	0.37 (0.047) ****	0.52 (0.062) ****	0.49 (0.089) ***	0.16 (0.022) ****	0.43 (0.058) ****
	Ref vs. x + 2	0.18 (0.024) ****	0.056 (0.016) **	0.22 (0.041) ***	0.24 (0.027) ****	0.21 (0.024) ****
	Ref vs. x + 4	0.53 (0.066) ****	0.37 (0.046) ****	0.59 (0.11) ***	0.60 (0.072) ****	0.90 (0.089) ****
	Ref vs. x + 6	0.62 (0.082) ****	0.53 (0.066) ****	0.70 (0.15) **	0.80 (0.096) ****	1.18 (0.13) ****
Canon Aquilion ONE	Ref vs. x − 6	−2.43 (0.25) ****	−1.65 (0.16) ****	−2.70 (0.42) ****	−2.85 (0.31) ****	−2.54 (0.22) ****
	Ref vs. x − 4	−3.04 (0.31) ****	−2.50 (0.23) ****	−3.22 (0.47) ****	−3.06 (0.32) ****	−3.25 (0.29) ****
	Ref vs. x − 2	−2.84 (0.29) ****	−2.59 (0.24) ****	−2.95 (0.43) ****	−2.56 (0.26) ****	−3.03 (0.27) ****
	Ref vs. x + 2	−2.63 (0.27) ****	−2.88 (0.28) ****	−2.82 (0.40) ****	−1.74 (0.17) ****	−2.54 (0.24) ****
	Ref vs. x + 4	−2.66 (0.27) ****	−3.12 (0.30) ****	−3.00 (0.43) ****	−1.45 (0.14) ****	−2.31 (0.23) ****
	Ref vs. x + 6	−2.16 (0.23) ****	−2.75 (0.28) ****	−2.62 (0.39) ****	−0.81 (0.080) ****	−1.54 (0.18) ****
Philips Spectral CT 7500	Ref vs. x − 6	2.05 (0.22) ****	2.56 (0.26) ****	2.21 (0.35) ****	1.13 (0.13) ****	2.12 (0.24) ****
	Ref vs. x − 4	1.95 (0.21) ****	2.27 (0.23) ****	2.19 (0.34) ****	1.19 (0.13) ****	1.91 (0.21) ****
	Ref vs. x − 2	1.40 (0.16) ****	1.48 (0.15) ****	1.59 (0.25) ****	0.94 (0.11) ****	1.41 (0.15) ****
	Ref vs. x + 2	0.37 (0.059) ****	−0.00022 (0.048) ns	0.20 (0.050) **	0.40 (0.053) ****	1.17 (0.11) ****
	Ref vs. x + 4	1.87 (0.20) ****	1.58 (0.16) ****	2.03 (0.32) ****	1.84 (0.20) ****	2.35 (0.22) ****
	Ref vs. x + 6	2.17 (0.23) ****	1.84 (0.19) ****	2.21 (0.36) *****	2.20 (0.24) ****	2.94 (0.27) ****

**Table A7:** Results of the Bland–Altman comparison. Per scanner and organ, the bias indicates how much (dis)agreement in organ doses there is when horizontal off-centre positioning is preceded by either a single PA or dual PA+LAT localiser (single vs. dual). Values are the mean difference (bias) in percentage with the standard error of the bias between brackets. A positive bias means that the organ dose was bigger for the single PA localiser instead of the dual PA+LAT localiser. Significance codes: ****: *p* < 0.0001; ***: *p* < 0.001; **: *p* < 0.01; *: *p* < 0.05; ns (not significant): *p* ≥ 0.05.

Horizontal Off-Centring|Bland–Altman Results						
Scanner	Comparison	Organ				
		Lung	Heart	Breast	Liver	Thyroid
GE Revolution CT	x − 6: single vs. dual	10.70 (4.11) **	2.13 (6.29) ns	7.69 (10.11) ns	−10.64 (2.10) ***	36.6 (2.15) ****
	x − 4: single vs. dual	33.63 (5.82) ****	22.11 (8.09) **	30.21 (10.39) **	−6.84 (4.25) ns	54.03 (1.49) ****
	x − 2: single vs. dual	4.03 (1.87) *	−0.61 (1.38) ns	0.43 (4.65) ns	−0.46 (1.07) ns	20.98 (1.37) ****
	x + 2: single vs. dual	8.49 (0.99) ****	11.28 (0.77) ****	11.58 (2.73) ****	11.05 (0.62) ****	−3.33 (2.43) ns
	x + 4: single vs. dual	27.7 (4.36) ****	15.28 (4.82) **	21.46 (9.01) *	3.2 (2.69) ns	55.42 (2.05) ****
	x + 6: single vs. dual	13.2 (5.60) *	−3.13 (6.37) ns	4.11 (12.01) ns	−15.60 (2.07) ****	60.33 (4.57) ****
Siemens SOMATOM Definition Flash	x − 6: single vs. dual	−10.14 (1.29) ****	−11.48 (3.64) ns	−11.13 (2.02) ***	−14.27 (1.01) ****	−6.90 (1.00) ****
	x − 4: single vs. dual	−27.58 (1.50) ****	−30.44 (2.64) ****	−30.71 (2.61) ****	−32.26 (0.91) ****	−23.43 (5.09) **
	x − 2: single vs. dual	−20.88 (−13.98) ****	−21.33 (1.31) ****	−21.88 (1.76) ****	−17.06 (1.91) ****	−20.1 (1.62) ****
	x + 2: single vs. dual	−2.77 (1.37) ns	−6.03 (2.45) ns	−6.91 (3.19) ns	2.93 (2.15) ns	10.54 (1.59) ****
	x + 4: single vs. dual	0.29 (0.61) ns	−0.025 (1.66) ns	0.11 (0.66) ns	0.27 (0.53) ns	0.45 (0.83) ns
	x + 6: single vs. dual	16.23 (1.06) ****	13.82 (1.62) ****	13.92 (0.79) ****	13.17 (0.49) ****	18.71 (3.17) ****
Canon Aquilion ONE	x − 6: single vs. dual	3.93 (0.41) ****	3.97 (0.41) ****	3.90 (0.43) ****	3.86 (0.41) ****	3.86 (0.63) ****
	x − 4: single vs. dual	2.95 (0.52) ****	2.98 (0.53) ****	2.92 (0.65) ****	2.85 (0.48) ****	2.90 (0.64) ****
	x − 2: single vs. dual	−0.68 (0.41) ns	−0.67 (0.41) ns	−0.61 (0.41) ns	−0.67 (0.41) ns	−0.66 (0.45) ns
	x + 2: single vs. dual	3.68 (0.39) ****	3.66 (0.39) ****	3.68 (0.45) ****	3.74 (0.37) ****	3.73 (0.40) ****
	x + 4: single vs. dual	2.60 (0.64) ****	2.65 (0.85) **	2.90 (1.29) *	2.68 (0.83) **	2.46 (0.94) **
	x + 6: single vs. dual	3.72 (0.64) ****	3.68 (0.69) ****	3.38 (1.09) **	3.96 (0.63) ****	3.68 (0.54) ****
Philips Spectral CT 7500	x − 6: single vs. dual	−17.77 (0.46) ****	−16.9 (0.35) ****	−17.09 (0.48) ****	−16.92 (0.64) ****	−18.16 (0.87) ****
	x − 4: single vs. dual	−14.19 (0.79) ****	−12.12 (0.82) ****	−12.58 (1.39) ****	−10.8 (0.81) ****	−13.24 (1.51) ****
	x − 2: single vs. dual	−11.4 (0.98) ****	−9.66 (1.72) ***	−9.94 (2.57) ns	−8.05 (2.04) *	−5.97 (2.33) ns
	x + 2: single vs. dual	−2.38 (0.91) ns	−0.65 (0.77) ns	−0.59 (1.55) ns	3.10 (2.19) ns	−2.44 (2.00) ns
	x + 4: single vs. dual	−12.34 (0.65) ****	−10.73 (0.94) ****	−10.98 (1.95) ***	−7.42 (0.74) ****	−8.61 (1.53) ***
	x + 6: single vs. dual	−17.55 (0.74) ****	−19.13 (1.24) ****	−18.27 (1.48) ****	−18.14 (1.17) ****	−15.19 (0.53) ****
