# Supplementary material for: Influence of Off-Centre Positioning, Scan Direction, and Localiser Projection Angle on Organ-Specific Radiation Doses in Low-Dose Chest CT: A Simulation Study Across Four Scanner Models
Source: J Imaging. 2026 Mar 11;12(3):123. doi: 10.3390/jimaging12030123 (PMC13028044; doi:10.3390/jimaging12030123)
Supplement: Supplementary file 1 [file jimaging-12-00123-s001.zip › jimaging-4135251-supplementary.pdf]

Journal of
Imaging

MDPI

Supplementary Materials

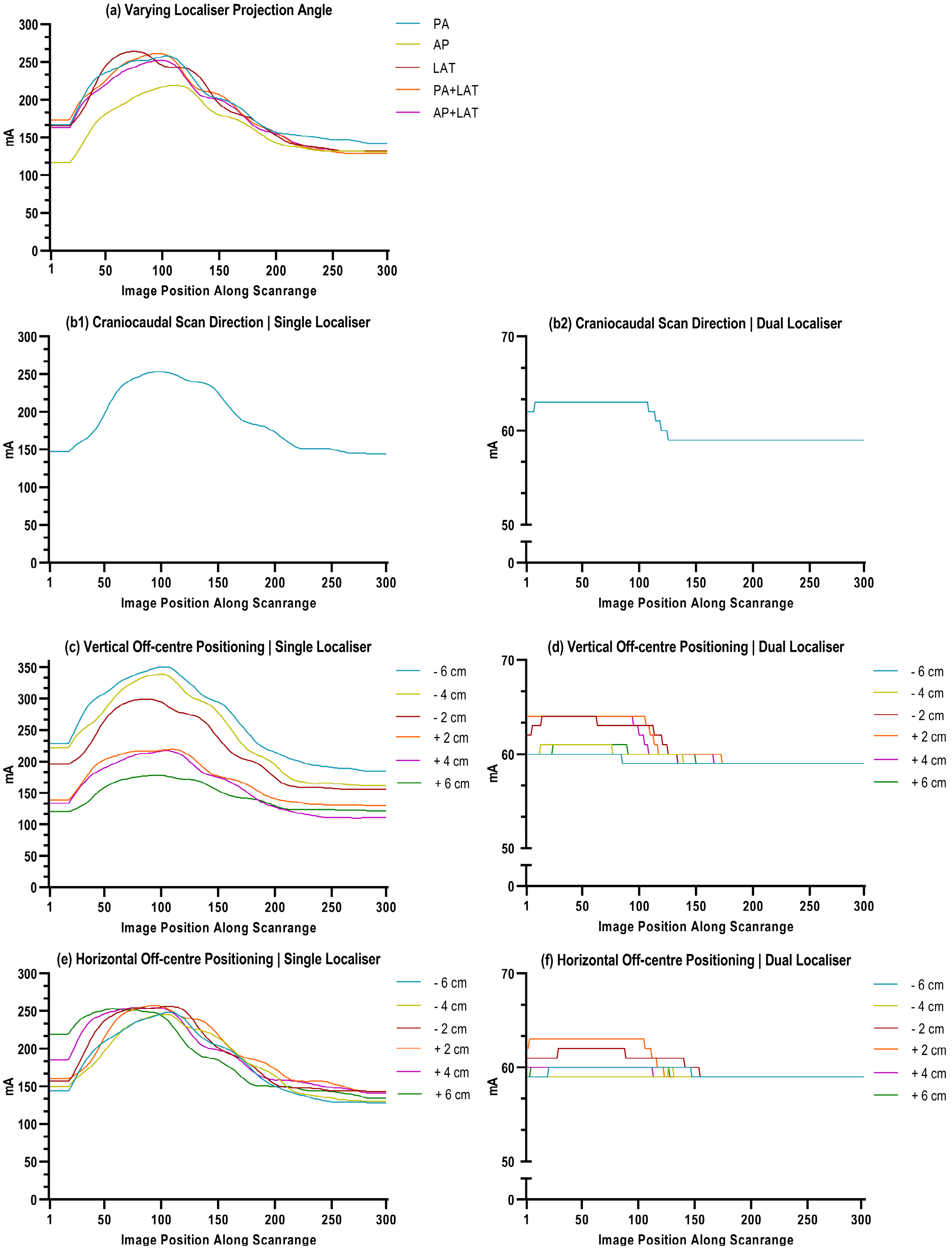

Figure S1: Automatic tube current modulation (ATCM) profiles derived from the experimental scanning setups with the RANDO phantom on the GE Revolution CT scanner. The tube current value (mA) is shown for every image position along the scanned range from the lung apex to the lung base for scans with a varying localiser projection angle (a), with a craniocaudal instead of caudocranial scan direction, preceded by a single (b1) or dual (b2) localiser, with vertical off-centre positioning, preceded by a single (c) or dual (d) localiser, or with horizontal off-centre positioning, preceded by

a single (e) or dual (f) localiser. For information: experiments corresponding to curves (a), (b1), (c) and (e) were acquired at a different time point than those for curves (b2), (d) and (f). Between the two time points, an adjustment had been made to the clinical scan protocol. The impact on the final Monte Carlo simulations was checked and minimised by re-acquiring an appropriate reference measurement for each deviating setup.

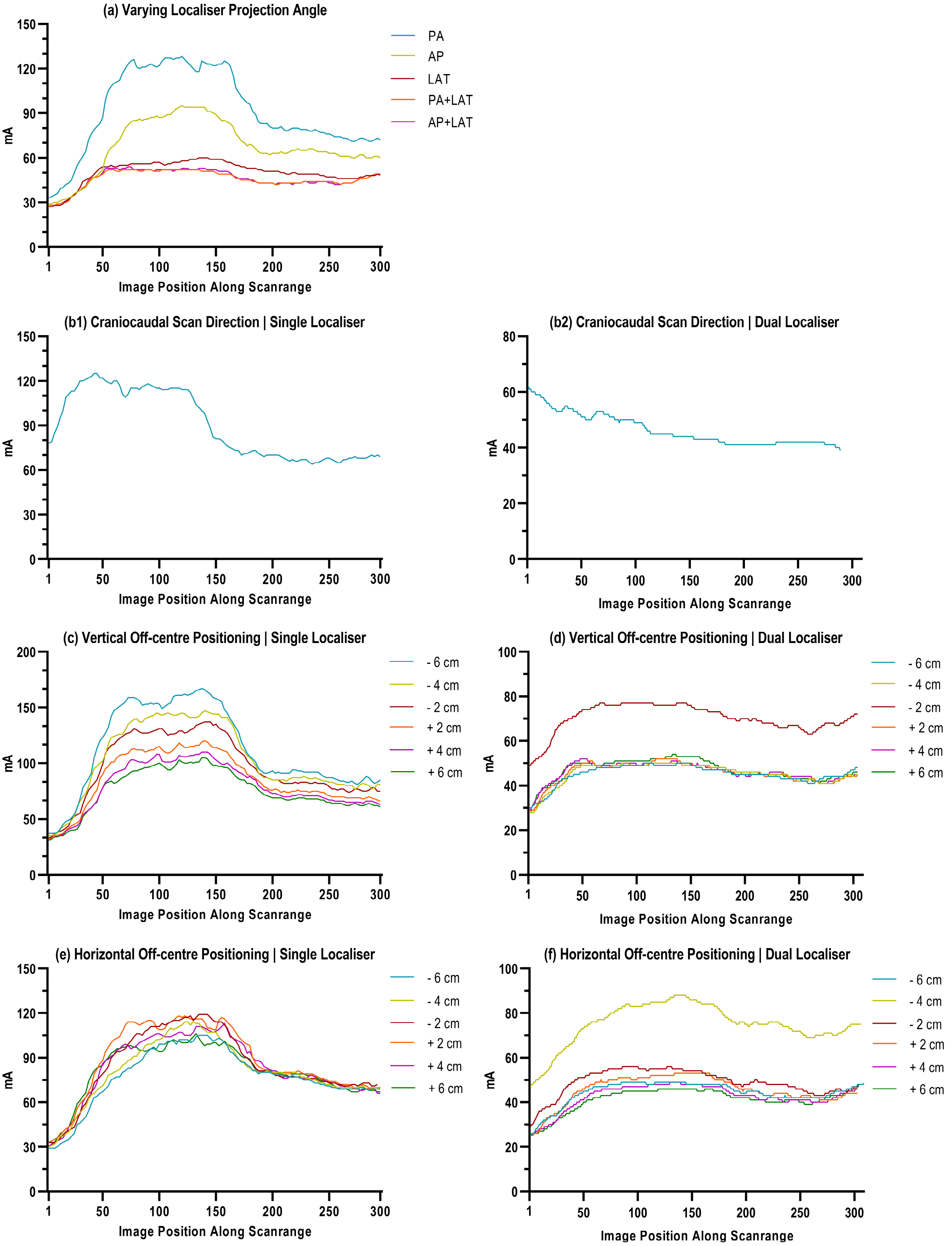

Figure S2: Automatic tube current modulation (ATCM) profiles derived from the experimental scanning setups with the RANDO phantom on the Siemens SOMATOM Definition Flash scanner. The tube current value (mA) is shown for every image position along the scanned range from the lung

apex to the lung base for scans with a varying localiser projection angle (a), with a craniocaudal instead of caudocranial scan direction, preceded by a single (b1) or dual (b2) localiser, with vertical off-centre positioning, preceded by a single (c) or dual (d) localiser, or with horizontal off-centre positioning, preceded by a single (e) or dual (f) localiser. For information: experiments corresponding to curves (a), (b1), (c) and (e) were acquired at a different time point than those for curves (b2), (d) and (f). Between the two time points, an adjustment had been made to the clinical scan protocol. The impact on the final Monte Carlo simulations was checked and minimised by re-acquiring an appropriate reference measurement for each deviating setup.

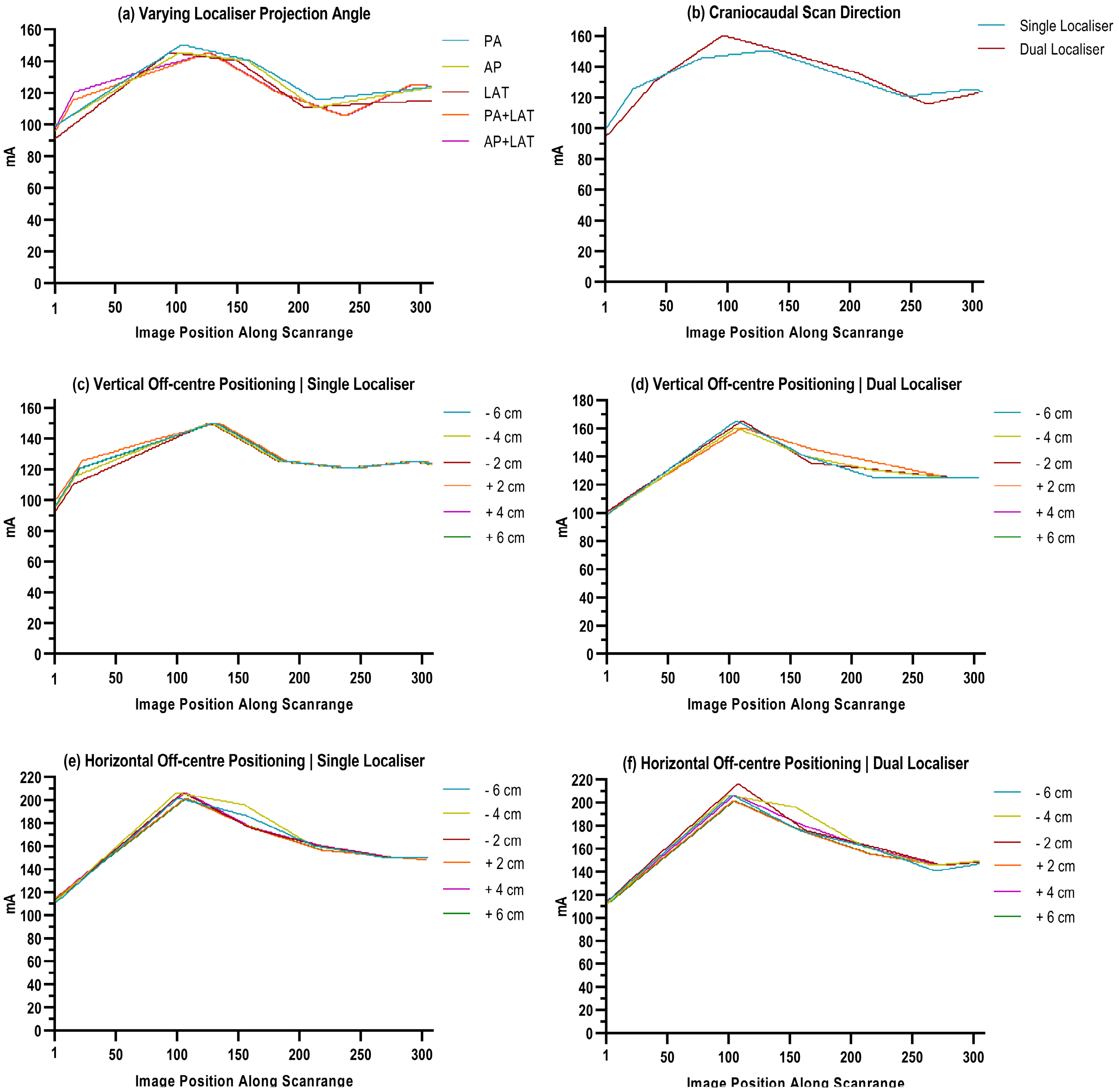

Figure S3: Automatic tube current modulation (ATCM) profiles derived from the experimental scanning setups with the RANDO phantom on the Canon Aquilion ONE scanner. The tube current value (mA) is shown for every image position along the scanned range from the lung apex to the lung base for scans with a varying localiser projection angle (a), with a craniocaudal instead of caudocranial scan direction, preceded by a single or dual localiser (b), with vertical off-centre positioning, preceded by a single (c) or dual (d) localiser, or with horizontal off-centre positioning, preceded by a single (e) or dual (f) localiser. All deviating setups were measured at the same point in time.

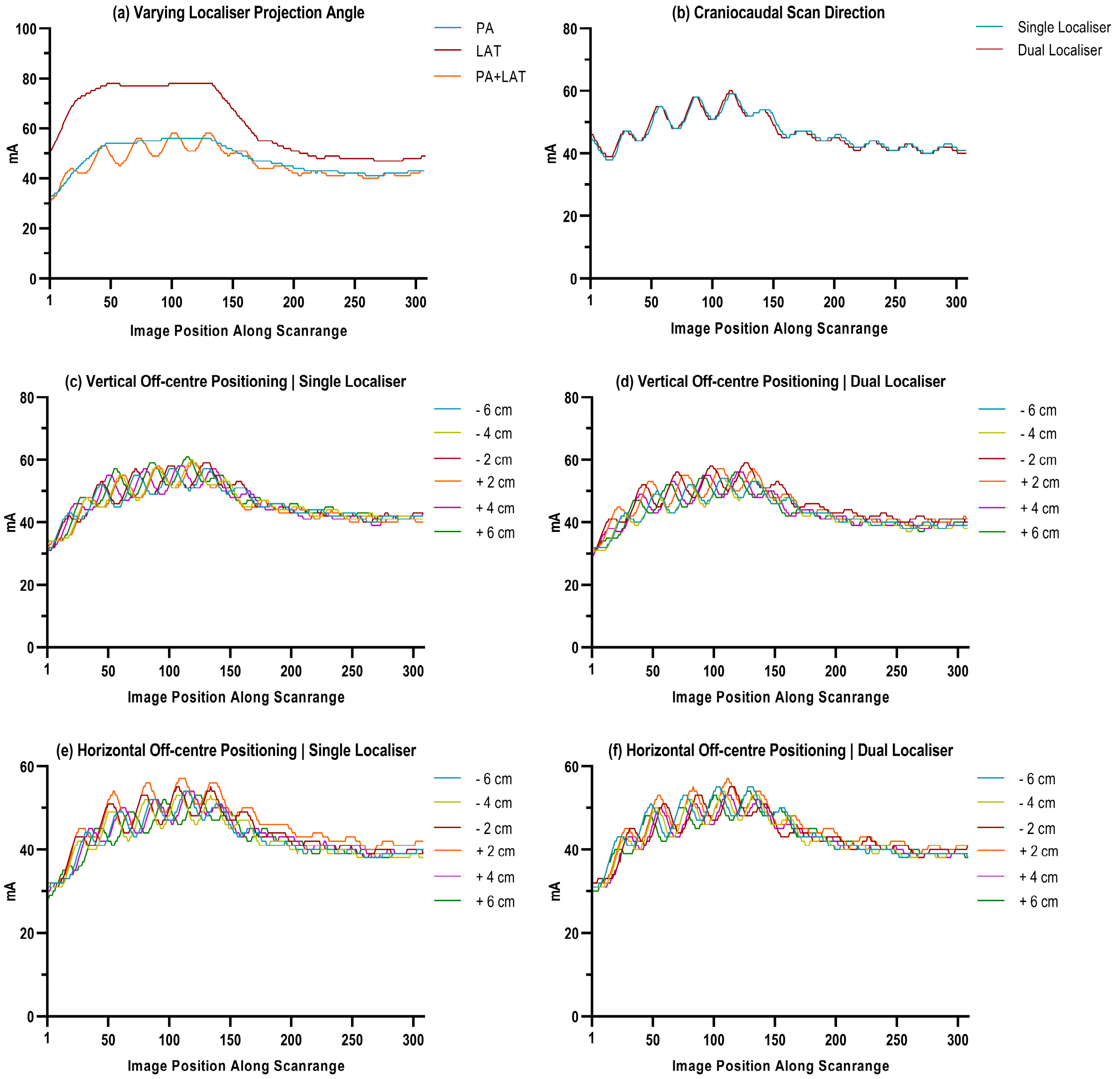

Figure S4: Automatic tube current modulation (ATCM) profiles derived from the experimental scanning setups with the RANDO phantom on the Philips Spectral CT 7500 scanner. The tube current value (mA) is shown for every image position along the scanned range from the lung apex to the lung base for scans with a varying localiser projection angle (a), with a craniocaudal instead of caudocranial scan direction, preceded by a single or dual localiser (b), with vertical off-centre positioning, preceded by a single (c) or dual (d) localiser, or with horizontal off-centre positioning, preceded by a single (e) or dual (f) localiser. All deviating setups were measured at the same point in time.

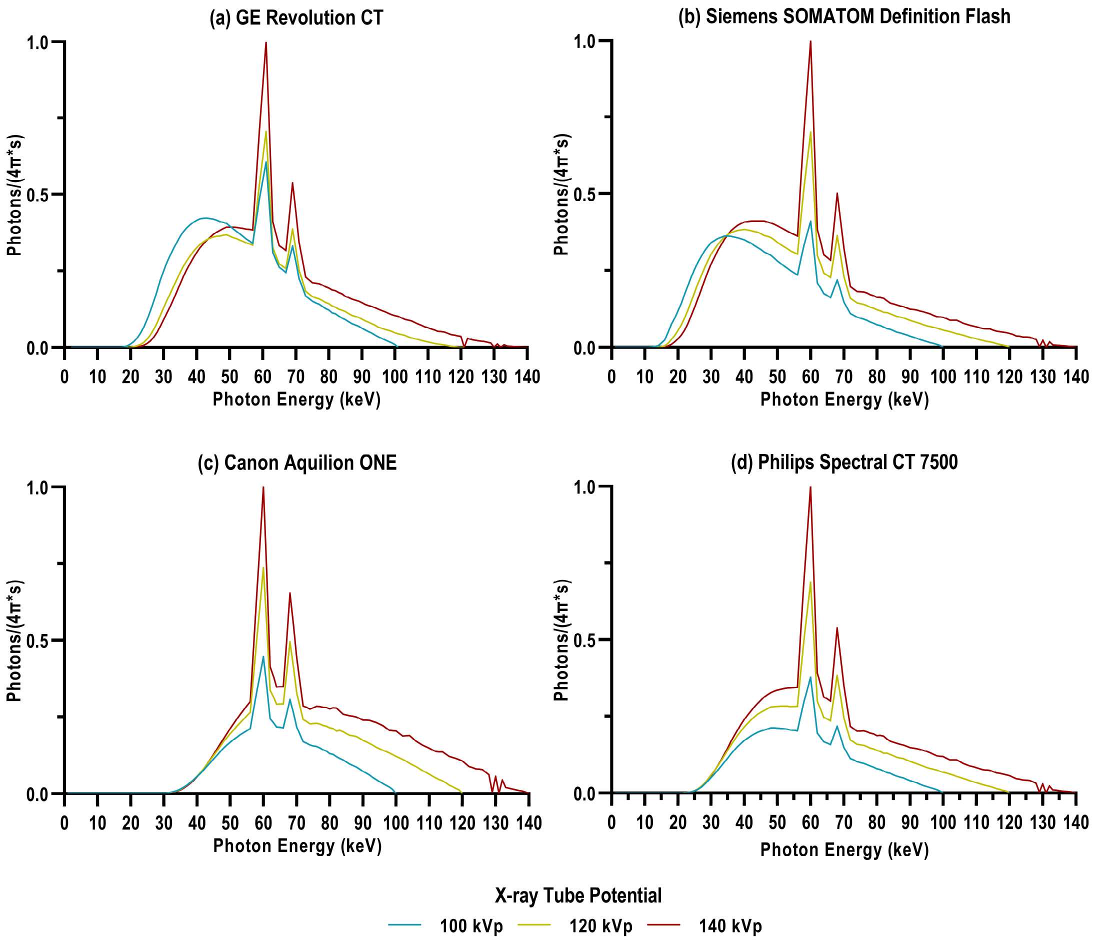

Figure S5: Generated x-ray spectra used as scanner-specific input for Monte Carlo dose simulations. The x-ray spectrum at tube voltages of 100, 120 and 140 kVp of each scanner are generated using MATLAB code with added SPEKTR tool, based on half-value layer measurements using a calibrated pencil beam ionisation chamber for four different CT scanner models: (a) GE Revolution CT, (b) Siemens SOMATOM Definition Flash, (c) Canon Aquilion One, (d) Philips Spectral CT 7500. SPEKTR generates the average emission of photons per unit of solid angle (isotropic x-ray source) which serves as the input for the ImpactMC Monte Carlo simulation software to model a CT scanner. Each graph is peak-normalised.

Table S1: Overview of the absolute organ doses from Monte Carlo simulations where the CT scan is preceded by a localiser from varying angles. Values are the mean dose in mGy of all voxelmodels within the according BMI class, with standard deviations between brackets. BMI classes 1-4 correspond respectively with voxel models that are underweight, normal weight, overweight and obese. Mind that the breast dose was only calculated for the female patients. (Abbreviations: PA: Posteroanterior; AP: Anteroposterior; LAT: Lateral).

Varying localiser projection angle							
**Scanner**	**Angle**	**BMI class**	**Organ**				
			**Lung**	**Heart**	**Breast**	**Liver**	**Thyroid**
GE Revolution CT	PA	1	1.95 (0.44)	2.28 (0.29)	2.52 (0.31)	1.55 (0.32)	3.04 (1.50)
		2	4.63 (1.51)	4.65 (1.03)	5.35 (2.09)	3.85 (1.40)	6.66 (2.54)
		3	5.89 (0.61)	5.75 (0.88)	6.79 (0.51)	4.64 (0.79)	6.08 (2.00)
		4	11.62 (1.18)	11.50 (1.36)	13.86 (0.19)	9.00 (1.31)	11.13 (2.66)
	AP	1	1.38 (0.31)	1.62 (0.20)	1.78 (0.21)	1.09 (0.22)	2.14 (1.05)
		2	3.28 (1.06)	3.29 (0.73)	3.78 (1.48)	2.73 (1.00)	4.72 (1.80)
		3	4.18 (0.43)	4.08 (0.62)	4.82 (0.37)	3.29 (0.56)	4.31 (1.43)
		4	8.22 (0.82)	8.14 (0.94)	9.83 (0.14)	6.37 (0.92)	7.90 (1.91)
	LAT	1	1.88 (0.44)	2.14 (0.27)	2.42 (0.25)	1.41 (0.29)	2.84 (1.37)
		2	4.49 (1.48)	4.37 (0.98)	5.04 (1.93)	3.51 (1.28)	6.25 (2.40)
		3	5.70 (0.59)	5.45 (0.84)	6.66 (0.47)	4.25 (0.73)	5.72 (1.93)
		4	11.20 (1.12)	10.86 (1.25)	13.37 (0.15)	8.25 (1.19)	10.47 (2.46)
	PA+LAT	1	1.88 (0.43)	2.14 (0.27)	2.36 (0.27)	1.41 (0.29)	3.16 (1.59)
		2	4.51 (1.48)	4.42 (0.96)	5.14 (1.98)	3.54 (1.29)	6.94 (2.63)
		3	5.78 (0.61)	5.58 (0.91)	6.80 (0.61)	4.29 (0.74)	6.35 (2.04)
		4	11.36 (1.12)	11.16 (1.30)	13.63 (0.34)	8.34 (1.20)	11.64 (2.94)
	AP+LAT	1	1.78 (0.40)	2.08 (0.26)	2.30 (0.26)	1.41 (0.29)	2.76 (1.35)
		2	4.22 (1.37)	4.24 (0.94)	4.86 (1.90)	3.51 (1.28)	6.09 (2.32)
		3	5.37 (0.55)	5.24 (0.80)	6.17 (0.46)	4.23 (0.71)	5.54 (1.82)
		4	10.58 (1.06)	10.47 (1.22)	12.64 (0.18)	8.19 (1.18)	10.17 (2.45)
Siemens SOMATOM Definition Flash	PA	1	0.95 (0.39)	2.28 (0.29)	2.52 (0.31)	1.55 (0.32)	3.04 (1.50)
		2	2.72 (0.87)	4.65 (1.03)	5.35 (2.09)	3.85 (1.40)	6.66 (2.54)
		3	3.43 (0.34)	5.75 (0.88)	6.79 (0.51)	4.64 (0.79)	6.08 (2.00)
		4	7.89 (0.91)	11.50 (1.36)	13.86 (0.19)	9.00 (1.31)	11.13 (2.66)
	AP	1	0.68 (0.28)	1.62 (0.20)	1.78 (0.21)	1.09 (0.22)	2.14 (1.05)
		2	1.95 (0.63)	3.29 (0.73)	3.78 (1.48)	2.73 (1.00)	4.72 (1.80)
		3	2.46 (0.25)	4.08 (0.62)	4.82 (0.37)	3.29 (0.56)	4.31 (1.43)
		4	5.66 (0.66)	8.14 (0.94)	9.83 (0.14)	6.37 (0.92)	7.90 (1.91)
	LAT	1	0.49 (0.20)	2.14 (0.27)	2.42 (0.25)	1.41 (0.29)	2.84 (1.37)
		2	1.40 (0.45)	4.37 (0.98)	5.04 (1.93)	3.51 (1.28)	6.25 (2.40)
		3	1.77 (0.18)	5.45 (0.84)	6.66 (0.47)	4.25 (0.73)	5.72 (1.93)
		4	4.06 (0.47)	10.86 (1.25)	13.37 (0.15)	8.25 (1.19)	10.47 (2.46)
	PA+LAT	1	0.44 (0.18)	2.14 (0.27)	2.36 (0.27)	1.41 (0.29)	3.16 (1.59)
		2	1.26 (0.40)	4.42 (0.96)	5.14 (1.98)	3.54 (1.29)	6.94 (2.63)
		3	1.59 (0.16)	5.58 (0.91)	6.80 (0.61)	4.29 (0.74)	6.35 (2.04)
		4	3.66 (0.43)	11.16 (1.30)	13.63 (0.34)	8.34 (1.20)	11.64 (2.94)
	AP+LAT	1	0.45 (0.18)	2.08 (0.26)	2.30 (0.26)	1.41 (0.29)	2.76 (1.35)
		2	1.28 (0.41)	4.24 (0.94)	4.86 (1.90)	3.51 (1.28)	6.09 (2.32)
		3	1.61 (0.16)	5.24 (0.80)	6.17 (0.46)	4.23 (0.71)	5.54 (1.82)
		4	3.71 (0.44)	10.47 (1.22)	12.64 (0.18)	8.19 (1.18)	10.17 (2.45)
Canon Aquilion ONE	PA	1	1.94 (0.43)	2.28 (0.32)	2.16 (0.32)	1.55 (0.28)	2.61 (1.19)
		2	4.45 (1.47)	4.48 (1.11)	4.65 (1.82)	3.72 (1.35)	5.87 (2.23)
		3	6.01 (0.71)	5.95 (0.86)	6.22 (0.53)	4.75 (0.66)	5.71 (1.90)

		4	10.22 (1.18)	10.06 (1.23)	10.67 (0.36)	7.73 (1.46)	9.14 (1.98)
	AP	1	1.85 (0.43)	2.19 (0.30)	2.04 (0.31)	1.43 (0.28)	2.39 (1.17)
		2	4.20 (1.38)	4.23 (1.05)	4.39 (1.71)	3.52 (1.27)	5.54 (2.11)
		3	5.67 (0.66)	5.61 (0.81)	5.85 (0.47)	4.47 (0.62)	5.38 (1.79)
		4	9.66 (1.11)	9.51 (1.16)	10.09 (0.36)	7.30 (1.37)	8.63 (1.84)
	LAT	1	1.72 (0.38)	2.02 (0.28)	1.92 (0.30)	1.37 (0.25)	2.33 (1.05)
		2	3.95 (1.29)	3.98 (0.98)	4.11 (1.60)	3.31 (1.20)	5.21 (1.97)
		3	5.34 (0.65)	5.28 (0.78)	5.51 (0.48)	4.22 (0.60)	5.07 (1.69)
		4	9.08 (1.06)	8.94 (1.11)	9.47 (0.32)	6.87 (1.31)	8.13 (1.75)
	PA+LAT	1	1.77 (0.40)	2.01 (0.29)	1.90 (0.29)	1.47 (0.27)	2.63 (1.24)
		2	4.06 (1.34)	3.98 (0.99)	4.12 (1.62)	3.56 (1.26)	5.95 (2.25)
		3	5.51 (0.66)	5.30 (0.76)	5.56 (0.47)	4.49 (0.57)	5.69 (1.96)
		4	9.28 (1.04)	8.96 (1.06)	9.44 (0.30)	7.30 (1.29)	9.04 (1.97)
	AP+LAT	1	1.81 (0.41)	2.02 (0.29)	1.91 (0.30)	1.47 (0.27)	2.83 (1.33)
		2	4.15 (1.38)	4.00 (1.01)	4.13 (1.62)	3.56 (1.27)	6.34 (2.42)
		3	5.63 (0.68)	5.34 (0.76)	5.73 (0.46)	4.48 (0.56)	6.12 (2.10)
		4	9.42 (1.10)	9.00 (1.09)	9.49 (0.37)	7.29 (1.31)	9.66 (2.23)
Philips Spectral CT 7500	PA	1	2.73 (0.58)	3.11 (0.45)	3.27 (0.49)	2.39 (0.56)	3.90 (1.80)
		2	5.75 (1.80)	5.59 (1.25)	6.59 (2.47)	4.58 (1.78)	7.89 (2.87)
		3	8.00 (0.89)	7.68 (1.26)	9.04 (1.02)	6.55 (0.41)	7.68 (2.29)
		4	14.51 (1.61)	13.95 (1.78)	17.48 (1.00)	11.35 (2.69)	13.44 (3.24)
	LAT	1	4.72 (1.00)	5.38 (0.78)	5.65 (0.85)	4.14 (0.98)	6.76 (3.14)
		2	10.26 (3.28)	9.91 (2.28)	11.40 (4.30)	8.17 (2.04)	14.07 (4.18)
		3	13.84 (1.53)	13.31 (2.19)	15.67 (1.77)	11.33 (0.73)	13.29 (3.93)
		4	25.10 (2.75)	24.13 (3.07)	30.25 (1.68)	19.64 (2.49)	21.77 (2.60)
	PA+LAT	1	2.56 (0.55)	2.89 (0.42)	3.09 (0.45)	2.19 (0.52)	3.80 (1.83)
		2	5.53 (1.77)	5.31 (1.24)	6.07 (2.31)	4.31 (1.74)	7.99 (3.03)
		3	7.51 (0.84)	7.22 (1.14)	8.63 (1.01)	6.01 (0.38)	7.44 (2.26)
		4	13.65 (1.51)	13.19 (1.76)	16.38 (1.04)	10.44 (2.50)	13.01 (3.16)

https://doi.org/10.3390/jimaging12030123

Table S2: Overview of the absolute organ doses from Monte Carlo simulations where the CT scan direction is changed to craniocaudal, preceded by either a single PA or dual PA+LAT localiser. Values are the mean dose in mGy of all voxelmodels within the according BMI class, with standard deviations between brackets. BMI classes 1-4 correspond respectively with voxel models that are underweight, normal weight, overweight and obese. Mind that the breast dose was only calculated for the female patients.

Absolute organ doses ∣ Varying CT scan direction after single PA localiser							
Scanner	CT scan direction	BMI class	Organ				
			Lung	Heart	Breast	Liver	Thyroid
GE Revolution CT	Craniocaudal	1	2.12 (0.56)	2.67 (0.51)	2.99 (0.57)	1.82 (0.43)	2.63 (1.17)
		2	4.56 (1.41)	4.99 (1.09)	5.79 (2.28)	4.22 (1.53)	5.65 (2.15)
		3	5.81 (0.61)	6.08 (0.93)	6.79 (0.59)	5.05 (0.82)	5.18 (1.68)
		4	11.64 (1.26)	12.2 (1.50)	14.71 (0.62)	9.83 (1.46)	9.64 (2.29)
Siemens SOMATOM Definition Flash	Craniocaudal	1	0.80 (0.37)	0.81 (0.34)	1.12 (0.46)	0.53 (0.20)	1.85 (0.94)
		2	2.27 (0.74)	2.04 (0.49)	2.55 (1.04)	1.53 (0.44)	5.07 (1.97)
		3	2.87 (0.32)	2.52 (0.38)	3.57 (0.27)	1.95 (0.31)	4.30 (1.64)
		4	6.43 (0.73)	5.75 (0.76)	7.61 (0.40)	4.00 (1.00)	8.87 (2.42)
Canon Aquilion ONE	Craniocaudal	1	2.18 (0.49)	2.57 (0.37)	2.43 (0.38)	1.76 (0.32)	2.97 (1.37)
		2	4.78 (1.55)	4.88 (1.24)	4.83 (1.91)	4.03 (1.30)	6.38 (2.34)
		3	6.76 (0.79)	6.73 (0.97)	6.96 (0.56)	4.51 (0.74)	6.52 (2.18)
		4	11.48 (1.35)	11.40 (1.42)	12.12 (0.40)	8.82 (1.67)	10.41 (2.25)
Philips Spectral CT 7500	Craniocaudal	1	2.64 (0.56)	3.03 (0.45)	3.18 (0.50)	2.36 (0.56)	4.04 (1.89)
		2	5.72 (1.82)	5.62 (1.26)	6.49 (2.44)	4.65 (1.89)	8.26 (3.11)
		3	7.73 (0.86)	7.49 (1.31)	8.67 (0.96)	6.52 (0.44)	7.90 (2.17)
		4	14.06 (1.56)	13.60 (1.69)	17.09 (0.89)	11.24 (2.69)	13.91 (3.45)

Absolute organ doses ∣ Varying CT scan direction after dual localiser							
Scanner	CT scan direction	BMI class	Organ				
			Lung	Heart	Breast	Liver	Thyroid
GE Revolution CT	Craniocaudal	1	1.82 (0.38)	2.18 (0.30)	2.37 (0.31)	1.50 (0.31)	2.59 (1.30)
		2	4.36 (1.40)	4.49 (0.99)	5.12 (2.03)	3.80 (1.38)	5.95 (2.25)
		3	5.71 (0.52)	5.66 (0.76)	6.73 (0.21)	4.71 (0.84)	5.48 (1.69)
		4	11.02 (1.15)	11.03 (1.33)	13.05 (0.53)	8.86 (1.32)	9.98 (2.42)
Siemens SOMATOM Definition Flash	Craniocaudal	1	0.67 (0.16)	0.68 (0.14)	0.87 (0.16)	0.48 (0.11)	1.63 (0.89)
		2	2.15 (0.69)	1.92 (0.48)	2.42 (1.01)	1.47 (0.44)	5.01 (1.85)
		3	2.83 (0.45)	2.41 (0.53)	3.38 (0.36)	1.92 (0.40)	4.40 (1.59)
		4	6.14 (0.65)	5.43 (0.70)	7.27 (0.34)	3.79 (0.99)	9.00 (2.42)
Canon Aquilion ONE	Craniocaudal	1	1.97 (0.44)	2.31 (0.30)	2.17 (0.34)	1.47 (0.27)	2.54 (1.14)
		2	4.54 (1.48)	4.55 (1.11)	4.63 (1.74)	3.56 (1.29)	5.76 (2.21)
		3	6.19 (0.78)	6.08 (0.93)	6.23 (0.59)	4.64 (0.69)	5.89 (1.91)
		4	10.39 (1.21)	10.21 (1.29)	10.90 (0.45)	7.49 (1.43)	8.92 (1.87)
Philips Spectral CT 7500	Craniocaudal	1	2.76 (0.59)	3.16 (0.48)	3.29 (0.53)	2.42 (0.57)	4.14 (1.89)
		2	5.97 (1.89)	5.83 (1.31)	6.70 (2.49)	4.72 (.92)	8.39 (3.16)
		3	8.08 (0.87)	7.75 (1.31)	9.05 (1.01)	6.65 (0.44)	8.08 (2.24)
		4	14.64 (1.59)	14.00 (1.69)	17.66 (0.84)	11.46 (2.70)	14.12 (3.42)

Table S3: Overview of the absolute organ doses from Monte Carlo simulations where the voxel models were positioned off-centre in the vertical direction, preceded by either a single PA or dual PA+LAT localiser. Positions are depicted as negative or positive deviations, respectively being too high or low with respect to the scanner isocentre. Values are the mean dose in mGy of all voxelmodels within the according BMI class, with standard deviations between brackets. BMI classes 1-4 correspond respectively with voxel models that are underweight, normal weight, overweight and obese. Mind that the breast dose was only calculated for the female patients.

**Absolute organ doses ǀ Vertical off-centring with single PA localiser**							
**Scanner**	**Position deviation**	**BMI class**	**Organ**				
			**Lung**	**Heart**	**Breast**	**Liver**	**Thyroid**
GE Revolution CT	- 6 cm	1	2.97 (0.77)	3.69 (0.61)	4.67 (0.84)	2.38 (0.51)	4.73 (2.05)
		2	6.42 (2.13)	6.74 (1.49)	8.33 (3.06)	5.37 (1.96)	9.60 (3.85)
		3	8.36 (0.74)	8.85 (1.58)	11.77 (1.02)	6.88 (1.19)	9.00 (2.62)
		4	17.00 (2.37)	18.63 (3.27)	24.47 (0.87)	13.91 (2.84)	17.00 (4.10)
	- 4 cm	1	2.84 (0.74)	3.47 (0.60)	4.26 (0.78)	2.27 (0.50)	4.42 (1.95)
		2	6.21 (2.06)	6.48 (1.42)	7.86 (3.01)	5.21 (1.89)	9.28 (3.65)
		3	7.97 (0.72)	8.28 (1.41)	10.56 (0.88)	6.51 (1.11)	8.52 (2.58)
		4	16.20 (1.98)	17.27 (2.57)	22.12 (0.30)	13.07 (2.34)	16.09 (3.86)
	- 2 cm	1	2.54 (0.68)	3.03 (0.57)	3.62 (0.70)	2.02 (0.47)	3.85 (1.72)
		2	5.52 (1.80)	5.68 (1.24)	6.70 (2.57)	4.63 (1.68)	8.14 (3.15)
		3	7.09 (0.68)	7.17 (1.15)	8.78 (0.69)	5.71 (0.95)	7.47 (2.38)
		4	14.16 (1.60)	14.58 (1.96)	18.05 (0.26)	11.21 (1.83)	13.87 (3.37)
	+ 2 cm	1	1.66 (0.47)	1.85 (0.39)	2.07 (0.42)	1.28 (0.33)	2.37 (1.09)
		2	3.68 (1.21)	3.59 (0.82)	4.00 (1.59)	3.03 (1.12)	5.16 (1.94)
		3	4.65 (0.51)	4.36 (0.64)	5.02 (0.39)	3.57 (0.62)	4.69 (1.59)
		4	9.11 (0.83)	8.64 (0.90)	10.23 (0.20)	6.90 (0.90)	8.58 (2.13)
	+ 4 cm	1	1.47 (0.42)	1.58 (0.35)	1.72 (0.35)	1.11 (0.31)	2.02 (0.93)
		2	3.32 (1.11)	3.12 (0.75)	3.40 (1.37)	2.69 (1.01)	4.53 (1.69)
		3	4.17 (0.50)	3.75 (0.54)	4.26 (0.37)	3.11 (0.57)	4.12 (1.45)
		4	8.10 (0.66)	7.37 (0.69)	8.61 (0.21)	5.99 (0.73)	7.46 (1.84)
	+ 6 cm	1	1.13 (0.33)	1.15 (0.27)	1.25 (0.26)	0.83 (0.25)	1.49 (0.69)
		2	2.58 (0.88)	2.34 (0.59)	2.49 (1.00)	2.06 (0.80)	3.41 (1.27)
		3	3.24 (0.42)	2.80 (0.42)	3.19 (0.28)	2.35 (0.45)	3.13 (1.11)
		4	6.26 (0.48)	5.46 (0.49)	6.35 (0.18)	4.52 (0.53)	5.66 (1.42)
Siemens SOMATOM Definition Flash	- 6 cm	1	1.07 (0.41)	1.26 (0.41)	1.90 (0.63)	0.84 (0.26)	1.71 (0.79)
		2	3.03 (0.99)	3.10 (0.74)	4.27 (1.65)	2.36 (0.63)	4.57 (1.87)
		3	3.91 (0.35)	4.01 (0.71)	5.87 (0.51)	3.23 (0.52)	4.09 (1.30)
		4	9.18 (1.35)	9.65 (1.89)	13.91 (1.01)	6.84 (2.08)	8.95 (2.22)
	- 4 cm	1	1.01 (0.40)	1.20 (0.40)	1.74 (0.59)	0.79 (0.25)	1.63 (0.77)
		2	2.88 (0.95)	2.96 (0.70)	4.01 (1.62)	2.25 (0.61)	4.40 (1.76)
		3	3.69 (0.35)	3.76 (0.64)	5.27 (0.46)	3.03 (0.50)	3.88 (1.29)
		4	8.59 (1.17)	8.84 (1.53)	12.38 (0.78)	6.30 (1.80)	8.39 (2.10)
	- 2 cm	1	0.99 (0.40)	1.15 (0.41)	1.63 (0.57)	0.77 (0.25)	1.56 (0.74)
		2	2.79 (0.89)	2.86 (0.65)	3.76 (1.51)	2.19 (0.60)	4.27 (1.67)
		3	3.55 (0.34)	3.54 (0.56)	4.81 (0.43)	2.87 (0.46)	3.73 (1.30)
		4	8.23 (1.03)	8.23 (1.24)	11.28 (0.71)	5.91 (1.59)	7.99 (1.99)
	+ 2 cm	1	0.89 (0.37)	0.97 (0.37)	1.30 (0.47)	0.67 (0.24)	1.28 (0.61)
		2	2.54 (0.82)	2.49 (0.61)	3.12 (1.31)	1.96 (0.58)	3.72 (1.44)
		3	3.20 (0.35)	2.98 (0.45)	3.89 (0.38)	2.47 (0.41)	3.26 (1.27)
		4	7.32 (0.80)	6.80 (0.82)	9.10 (0.64)	5.01 (1.21)	6.79 (1.68)
	+ 4 cm	1	0.82 (0.35)	0.85 (0.34)	1.15 (0.41)	0.60 (0.22)	1.12 (0.54)
		2	2.38 (0.78)	2.25 (0.58)	2.78 (1.18)	1.80 (0.56)	3.33 (1.28)

		3	2.96 (0.34)	2.66 (0.40)	3.48 (0.36)	2.23 (0.38)	2.93 (1.16)
		4	6.76 (0.68)	6.02 (0.65)	8.08 (0.59)	4.51 (1.03)	6.09 (1.54)
	+ 6 cm	1	0.76 (0.32)	0.75 (0.30)	1.04 (0.36)	0.55 (0.20)	0.99 (0.47)
		2	2.24 (0.75)	2.04 (0.55)	2.52 (1.06)	1.67 (0.53)	2.97 (1.13)
		3	2.80 (0.35)	2.41 (0.37)	3.19 (0.34)	2.05 (0.37)	2.68 (1.07)
		4	6.33 (0.62)	5.44 (0.57)	7.33 (0.52)	4.12 (0.92)	5.55 (1.41)
Canon Aquilion ONE	- 6 cm	1	1.73 (0.38)	2.18 (0.30)	2.19 (0.32)	1.51 (0.28)	2.59 (1.16)
		2	3.85 (1.27)	4.14 (1.03)	4.61 (1.78)	3.47 (1.25)	5.60 (2.20)
		3	5.30 (0.53)	5.72 (0.88)	6.58 (0.54)	4.62 (0.62)	5.48 (1.71)
		4	9.34 (1.47)	10.15 (1.73)	11.87 (0.29)	7.88 (1.85)	9.00 (1.97)
	- 4 cm	1	1.81 (0.40)	2.24 (0.32)	2.21 (0.32)	1.56 (0.28)	2.61 (1.18)
		2	4.06 (1.32)	4.31 (1.05)	4.70 (1.82)	3.64 (1.30)	5.74 (2.22)
		3	5.57 (0.60)	5.86 (0.89)	6.49 (0.50)	4.78 (0.65)	5.58 (1.81)
		4	9.67 (1.39)	10.20 (1.57)	11.52 (0.14)	8.00 (1.62)	9.07 (1.98)
	- 2 cm	1	1.86 (0.41)	2.28 (0.34)	2.18 (0.33)	1.60 (0.29)	2.54 (1.16)
		2	4.21 (1.37)	4.42 (1.09)	4.71 (1.86)	3.79 (1.36)	5.69 (2.18)
		3	5.73 (0.63)	5.89 (0.85)	6.25 (0.51)	4.88 (0.66)	5.48 (1.79)
		4	9.85 (1.26)	10.09 (1.37)	11.01 (0.16)	8.05 (1.62)	8.88 (1.93)
	+ 2 cm	1	2.04 (0.47)	2.31 (0.37)	2.11 (0.36)	1.68 (0.32)	2.82 (1.30)
		2	4.73 (1.58)	4.63 (1.20)	4.76 (1.96)	4.12 (1.52)	6.44 (2.44)
		3	6.34 (0.78)	6.01 (0.83)	6.07 (0.54)	5.08 (0.73)	6.23 (2.15)
		4	10.64 (1.10)	9.98 (1.03)	10.24 (0.53)	8.22 (1.40)	9.87 (2.20)
	+ 4 cm	1	1.97 (0.46)	2.18 (0.36)	1.97 (0.34)	1.60 (0.31)	2.54 (1.19)
		2	4.63 (1.56)	4.44 (1.18)	4.44 (1.84)	4.01 (1.49)	5.90 (2.22)
		3	6.19 (0.81)	5.70 (0.80)	5.44 (0.57)	4.87 (0.75)	5.71 (2.07)
		4	10.28 (0.99)	9.35 (0.89)	9.18 (0.39)	7.80 (1.28)	8.94 (1.99)
	+ 6 cm	1	1.96 (0.47)	2.10 (0.35)	1.86 (0.33)	1.55 (0.32)	2.43 (1.13)
		2	4.66 (1.59)	4.34 (1.19)	4.22 (1.76)	3.96 (1.50)	5.74 (2.16)
		3	6.21 (0.87)	5.51 (0.80)	5.08 (0.59)	4.73 (0.79)	5.60 (2.08)
		4	10.24 (0.92)	8.94 (0.77)	8.53 (0.36)	7.56 (1.18)	8.72 (1.99)
Philips Spectral CT 7500	- 6 cm	1	2.37 (0.49)	2.91 (0.39)	3.31 (0.49)	2.17 (0.57)	3.63 (1.71)
		2	5.06 (1.64)	5.22 (1.20)	6.36 (2.31)	4.06 (1.68)	7.27 (2.85)
		3	6.93 (0.67)	7.25 (1.26)	9.23 (0.95)	6.02 (0.49)	6.93 (1.90)
		4	12.91 (1.78)	13.68 (2.40)	18.25 (1.32)	10.80 (3.09)	12.37 (3.00)
	- 4 cm	1	2.46 (0.51)	2.95 (0.41)	3.26 (0.48)	2.23 (0.56)	3.51 (1.59)
		2	5.28 (1.70)	5.37 (1.19)	6.44 (2.34)	4.27 (1.76)	7.12 (2.73)
		3	7.20 (0.74)	7.34 (1.29)	9.00 (0.97)	6.20 (0.47)	6.85 (1.90)
		4	13.29 (1.73)	13.66 (2.09)	17.85 (0.91)	10.99 (2.96)	12.16 (2.82)
	- 2 cm	1	2.58 (0.54)	3.04 (0.43)	3.34 (0.49)	2.35 (0.57)	3.68 (1.75)
		2	5.55 (1.77)	5.56 (1.27)	6.55 (2.46)	4.58 (1.84)	7.66 (2.91)
		3	7.52 (0.80)	7.56 (1.24)	9.13 (1.01)	6.44 (0.41)	7.15 (2.14)
		4	13.80 (1.65)	13.94 (2.02)	17.77 (1.19)	11.28 (2.82)	12.63 (3.04)
	+ 2 cm	1	2.57 (0.56)	2.84 (0.44)	2.91 (0.46)	2.18 (0.51)	3.56 (1.66)
		2	5.60 (1.80)	5.30 (1.23)	5.93 (2.22)	4.33 (1.77)	7.44 (2.77)
		3	7.54 (0.89)	7.05 (1.18)	8.18 (0.97)	5.95 (0.42)	7.07 (2.08)
		4	13.61 (1.39)	12.70 (1.47)	15.79 (0.95)	10.28 (2.33)	12.34 (2.96)
	+ 4 cm	1	2.60 (0.58)	2.80 (0.44)	2.86 (0.47)	2.12 (0.49)	3.42 (1.63)
		2	5.73 (1.88)	5.26 (1.29)	5.71 (2.18)	4.31 (1.76)	7.40 (2.71)
		3	7.71 (0.94)	6.98 (1.12)	8.11 (1.01)	5.82 (0.43)	6.92 (2.15)
		4	13.89 (1.33)	12.54 (1.40)	15.34 (1.03)	10.05 (2.15)	12.08 (2.86)
	+ 6 cm	1	2.68 (0.61)	2.80 (0.47)	2.86 (0.54)	2.26 (0.50)	3.59 (1.69)
		2	6.00 (1.98)	5.46 (1.36)	5.92 (2.26)	4.66 (1.97)	7.71 (2.83)
		3	8.02 (1.05)	7.08 (1.24)	8.05 (1.01)	6.23 (0.54)	7.38 (2.22)

https://doi.org/10.3390/jimaging12030123

		4	14.35 (1.33)	12.67 (1.30)	15.59 (1.06)	10.66 (2.26)	12.85 (3.17)

Absolute organ doses | Vertical off-centring with dual localiser

Scanner	Position deviation	BMI class	Organ				
			Lung	Heart	Breast	Liver	Thyroid
GE Revolution CT	- 6 cm	1	1.24 (0.23)	1.84 (0.26)	1.95 (0.38)	1.33 (0.26)	1.56 (0.74)
		2	2.83 (0.85)	3.55 (0.78)	4.37 (1.72)	3.22 (1.17)	3.41 (1.37)
		3	3.71 (0.39)	4.47 (0.89)	5.17 (0.58)	4.11 (0.72)	3.23 (0.93-
		4	7.59 (1.15)	9.30 (1.77)	11.79 (1.31)	8.15 (1.68)	6.15 (1.44)
	- 4 cm	1	1.53 (0.30)	2.08 (0.27)	2.39 (0.31)	1.42 (0.28)	1.90 (0.90)
		2	3.54 (1.11)	4.09 (0.91)	4.90 (1.91)	3.49 (1.27)	4.25 (1.67)
		3	4.57 (0.45)	5.11 (0.85)	5.98 (0.39)	4.37 (0.74)	3.97 (1.21)
		4	9.28 (1.24)	10.46 (1.70)	13.11 (1.16)	8.60 (1.58)	7.49 (1.77)
	- 2 cm	1	1.85 (0.39)	2.22 (0.29)	2.51 (0.30)	1.48 (0.29)	2.78 (1.36)
		2	4.40 (1.44)	4.52 (1.00)	5.25 (.04)	3.71 (1.35)	6.32 (2.44)
		3	5.53 (0.68)	5.56 (0.96)	6.83 (0.38)	4.46 (0.79)	5.58 (1.88)
		4	11.21 (1.26)	11.43 (1.56)	13.92 (0.55)	8.93 (1.46)	10.57 (2.53)
	+ 2 cm	1	2.02 (0.45)	2.22 (0.32)	2.40 (0.33)	1.50 (0.32)	3.20 (1.63)
		2	4.92 (1.64)	4.73 (1.08)	5.33 (2.08)	3.90 (1.46)	7.39 (2.78)
		3	6.26 (0.68)	5.91 (1.03)	6.81 (0.59)	4.60 (0.76)	6.71 (2.24)
		4	12.21 (1.09)	11.57 (1.19)	13.72 (0.19)	8.92 (1.17)	12.29 (3.12)
	+ 4 cm	1	1.96 (0.45)	2.08 (0.31)	2.15 (0.33)	1.46 (0.33)	2.93 (1.49)
		2	4.86 (1.63)	4.54 (1.09)	4.95 (1.98)	3.88 (1.47)	6.97 (2.59)
		3	6.13 (0.73)	5.50 (0.81)	6.30 (0.61)	4.49 (0.80)	6.34 (2.15)
		4	11.87 (0.98)	10.79 (1.02)	12.62 (0.31)	8.64 (1.05)	11.49 (2.93)
	+ 6 cm	1	1.66 (0.37)	1.86 (0.29)	1.92 (0.32)	1.37 (0.32)	1.74 (0.85)
		2	4.06 (1.33)	4.05 (1.01)	4.34 (1.81)	3.73 (1.44)	4.28 (1.60)
		3	5.10 (0.65)	4.71 (0.66)	4.99 (0.48)	4.23 (0.79)	3.96 (1.38)
		4	10.00 (0.90)	9.18 (0.91)	10.54 (0.80)	8.13 (0.97)	7.24 (1.75)
Siemens SOMATOM Definition Flash	- 6 cm	1	0.70 (0.14)	0.83 (0.11)	1.14 (0.13)	0.59 (0.15)	1.21 (0.60)
		2	2.34 (0.79)	2.42 (0.60)	3.35 (1.35)	1.92 (0.55)	3.61 (1.46)
		3	2.97 (0.26)	3.07 (0.55)	4.43 (0.42)	2.56 (0.41)	3.21 (0.99)
		4	7.03 (1.02)	7.42 (1.44)	10.75 (0.77)	5.45 (1.64)	7.04 (1.78)
	- 4 cm	1	0.73 (0.15)	0.89 (0.12)	1.17 (0.14)	0.61 (0.15)	1.21 (0.61)
		2	2.44 (0.78)	2.56 (0.60)	3.43 (1.37)	1.97 (0.53)	3.64 (1.45)
		3	3.11 (0.29)	3.20 (0.55)	4.41 (0.41)	2.64 (0.44)	3.21 (1.07)
		4	7.29 (0.98)	7.58 (1.30)	10.67 (0.69)	5.50 (1.59)	6.98 (1.73)
	- 2 cm	1	0.78 (.16)	0.92 (0.13)	1.18 (0.15)	0.64 (0.16)	1.33 (0.67)
		2	2.62 (0.84)	2.68 (0.63)	3.49 (1.42)	2.11 (0.59)	4.06 (1.58)
		3	3.34 (0.32)	3.30 (0.51)	4.45 (0.39)	2.75 (0.43)	3.58 (1.21)
		4	7.73 (0.97)	7.68 (1.16)	10.67 (0.65)	5.66 (1.51)	7.69 (1.94)
	+ 2 cm	1	0.80 (0.17)	0.87 (0.13)	1.08 (0.14)	0.62 (0.16)	1.21 (0.61)
		2	2.72 (0.88)	2.66 (0.65)	3.36 (1.42)	2.10 (0.62)	3.94 (1.52)
		3	3.41 (0.37)	3.19 (0.48)	4.17 (0.41)	2.64 (0.44)	3.45 (1.32)
		4	7.82 (0.84)	7.26 (0.87)	9.75 (0.70)	5.34 (1.29)	7.21 (1.80)
	+ 4 cm	1	0.80 (0.18)	0.84 (0.14)	1.03 (0.15)	0.63 (0.16)	1.21 (0.61)
		2	2.76 (0.90)	2.61 (0.68)	3.22 (1.37)	2.14 (0.66)	4.01 (1.52)
		3	3.46 (0.40)	3.08 (1.46)	4.02 (0.40)	2.65 (0.46)	3.54 (1.41)
		4	7.88 (0.81)	7.00 (0.75)	9.41 (0.63)	5.34 (1.25)	7.36 (1.86)
	+ 6 cm	1	0.81 (0.18)	0.80 (0.13)	1.02 (0.15)	0.60 (0.16)	1.13 (0.56)
		2	2.84 (0.96)	2.57 (0.70)	3.17 (1.34)	2.12 (0.68)	3.82 (1.43)
		3	3.56 (0.45)	3.04 (0.46)	4.05 (0.42)	2.59 (0.46)	3.43 (1.34)
		4	8.06 (0.79)	6.88 (0.72)	9.29 (0.60)	5.23 (1.17)	7.18 (1.84)
Can	- 6 cm	1	1.66 (0.36)	2.07 (0.27)	2.11 (0.28)	1.37 (0.25)	2.40 (1.06)

		2	3.73 (1.24)	3.95 (0.96)	4.35 (1.60)	3.16 (1.13)	5.20 (2.04)
		3	5.15 (0.51)	5.52 (0.88)	6.46 (0.54)	4.26 (0.56)	5.14 (1.56)
		4	9.06 (1.43)	9.80 (1.72)	11.50 (0.26)	7.22 (1.72)	8.47 (1.81)
	- 4 cm	1	1.75 (0.38)	2.16 (0.30)	2.14 (0.31)	1.46 (0.26)	2.47 (1.12)
		2	3.91 (1.28)	4.13 (1.00)	4.48 (1.71)	3.39 (1.22)	5.40 (2.09)
		3	5.36 (0.57)	5.65 (0.87)	6.30 (0.51)	4.47 (0.59)	5.28 (1.66)
		4	9.33 (1.33)	9.88 (1.54)	11.19 (0.16)	7.47 (1.64)	8.61 (1.85)
	- 2 cm	1	1.86 (0.41)	2.23 (0.31)	2.16 (0.32)	1.52 (0.27)	2.60 (1.17)
		2	4.23 (1.39)	4.34 (1.06)	4.62 (1.79)	3.60 (1.30)	5.77 (2.21)
		3	5.76 (0.64)	5.85 (0.87)	6.34 (0.51)	4.66 (0.63)	5.64 (1.84)
		4	9.89 (1.28)	10.06 (1.39)	11.03 (0.24)	7.69 (1.58)	9.09 (1.98)
	+ 2 cm	1	1.96 (0.44)	2.30 (0.34)	2.10 (0.37)	1.58 (0.30)	2.48 (1.13)
		2	4.53 (1.48)	4.59 (1.15)	4.68 (1.86)	3.88 (1.43)	5.64 (2.12)
		3	6.08 (0.76)	5.93 (0.86)	5.80 (0.54)	4.85 (0.73)	5.50 (1.86)
		4	10.26 (1.06)	9.94 (1.05)	10.19 (0.37)	7.82 (1.40)	8.77 (1.91)
	+ 4 cm	1	1.96 (0.45)	2.22 (0.34)	2.00 (0.36)	1.55 (0.30)	2.41 (1.10)
		2	4.57 (1.51)	4.51 (1.16)	4.48 (1.79)	3.87 (1.44)	5.55 (2.07)
		3	6.11 (0.81)	5.76 (0.83)	5.39 (0.56)	4.75 (0.76)	5.43 (1.89)
		4	10.23 (0.97)	9.54 (1.05)	9.45 (0.35)	7.61 (1.29)	8.61 (1.91)
	+ 6 cm	1	1.92 (0.46)	2.11 (0.34)	1.86 (0.34)	1.49 (0.31)	2.30 (1.05)
		2	4.56 (1.53)	4.36 (1.17)	4.23 (1.72)	3.80 (1.44)	5.39 (2.00)
		3	6.07 (0.85)	5.51 (0.81)	4.97 (0.58)	4.59 (0.79)	5.31 (1.88)
		4	10.07 (0.89)	9.03 (0.79)	8.64 (0.30)	7.30 (1.21)	8.36 (1.89)
Philips Spectral CT 7500	- 6 cm	1	2.18 (0.45)	2.55 (0.34)	2.87 (0.37)	1.89 (0.48)	3.31 (1.45)
		2	4.67 (1.55)	4.59 (1.06)	5.58 (2.02)	3.60 (1.47)	6.58 (2.54)
		3	6.45 (0.63)	6.45 (1.09)	8.47 (0.95)	5.27 (0.40)	6.39 (1.73)
		4	11.95 (1.65)	12.18 (2.12)	16.22 (0.77)	9.47 (2.64)	11.35 (2.61)
	- 4 cm	1	2.23 (0.46)	2.60 (0.35)	2.87 (0.38)	1.95 (0.48)	3.28 (1.46)
		2	4.80 (1.56)	4.71 (1.07)	5.62 (2.06)	3.74 (1.51)	6.67 (2.55)
		3	6.57 (0.66)	6.51 (1.09)	8.25 (0.90)	5.39 (0.38)	6.36 (1.82)
		4	12.09 (1.54)	12.13 (1.89)	15.80 (0.69)	9.55 (2.54)	11.26 (2.57)
	- 2 cm	1	2.67 (0.56)	3.14 (0.44)	3.36 (0.49)	2.35 (0.57)	3.79 (1.73)
		2	5.74 (1.84)	5.70 (1.28)	6.68 (2.44)	4.54 (1.85)	7.78 (2.97)
		3	7.81 (0.82)	7.74 (1.30)	9.28 (1.01)	6.48 (0.43)	7.43 (2.18)
		4	14.27 (1.70)	14.18 (1.98)	18.08 (0.89)	11.37 (2.87)	13.03 (3.02)
	+ 2 cm	1	2.65 (0.58)	2.91 (0.44)	2.99 (0.47)	2.23 (0.52)	3.66 (1.70)
		2	5.80 (1.87)	5.43 (1.27)	6.08 (2.30)	4.48 (1.81)	7.74 (2.86)
		3	7.83 (0.92)	7.25 (1.19)	8.44 (1.00)	6.13 (0.41)	7.29 (2.21)
		4	14.11 (1.43)	13.05 (1.52)	16.16 (0.94)	10.56 (2.38)	12.69 (2.98)
	+ 4 cm	1	2.55 (0.56)	2.72 (0.43)	2.71 (0.45)	2.09 (0.48)	3.33 (1.57)
		2	5.61 (1.83)	5.13 (1.22)	5.63 (2.12)	4.24 (1.74)	7.15 (2.64)
		3	7.54 (0.93)	6.77 (1.16)	7.86 (0.98)	5.75 (0.43)	6.74 (2.10)
		4	13.52 (1.28)	12.12 (1.28)	14.99 (0.87)	9.90 (2.13)	11.71 (2.78)
	+ 6 cm	1	2.47 (0.55)	2.50 (0.41)	2.52 (0.43)	1.93 (0.43)	3.14 (1.48)
		2	5.49 (1.83)	4.81 (1.21)	5.20 (1.96)	4.02 (1.68)	6.86 (2.54)
		3	7.39 (0.97)	6.36 (1.09)	7.49 (0.95)	5.39 (0.44)	6.49 (2.08)
		4	13.17 (1.20)	11.36 (1.16)	14.02 (0.95)	9.27 (1.94)	11.27 (2.68)

Table S4. Overview of the absolute organ doses from Monte Carlo simulations where the voxel models were positioned off-centre in the horizontal/lateral direction, preceded by either a single PA or dual PA+LAT localiser. Positions are depicted as negative or positive deviations, respectively being deviations to the participant's left-hand or right-hand side with respect to centreline laser.

Values are the mean dose in mGy of all voxelmodels within the according BMI class, with standard deviations between brackets. BMI classes 1-4 correspond respectively with voxel models that are underweight, normal weight, overweight and obese. Mind that the breast dose was only calculated for the female patients.

Absolute organ doses丨Horizontal off-centring with single PA localiser							
Scanner	Position deviation	BMI class	Organ				
			Lung	Heart	Breast	Liver	Thyroid
GE Revolution CT	- 6 cm	1	1.81 (0.50)	1.83 (0.40)	2.58 (0.51)	1.52 (0.40)	2.56 (1.20)
		2	4.04 (1.28)	3.46 (0.75)	5.06 (1.83)	3.69 (1.38)	5.33 (2.12)
		3	5.16 (0.47)	4.48 (0.78)	6.35 (0.61)	4.55 (0.81)	4.96 (1.54)
		4	10.14 (1.19)	8.92 (1.16)	13.37 (0.54)	8.71 (1.33)	8.85 (1.85)
	- 4 cm	1	1.96 (0.55)	2.24 (0.48)	2.87 (0.57)	1.53 (0.40)	2.66 (1.25)
		2	4.29 (1.33)	4.12 (0.91)	5.31 (1.92)	3.69 (1.38)	5.56 (2.17)
		3	5.47 (0.55)	5.16 (0.84)	6.56 (0.59)	4.54 (0.80)	5.15 (1.61)
		4	10.86 (1.26)	10.23 (1.36)	14.09 (0.54)	8.77 (1.33)	9.28 (2.00)
	- 2 cm	1	2.08 (0.58)	2.32 (0.48)	2.84 (0.56)	1.70 (0.42)	3.03 (1.36)
		2	4.61 (1.49)	4.37 (0.97)	5.25 (2.01)	4.00 (1.45)	6.46 (2.50)
		3	5.84 (0.58)	5.47 (0.3)	6.75 (0.46)	4.84 (0.80)	5.97 (1.96)
		4	11.49 (1.21)	10.82 (1.29)	13.87 (0.24)	9.29 (1.33)	10.75 (2.45)
	+ 2 cm	1	2.15 (0.57)	2.69 (0.50)	2.95 (0.58)	1.71 (0.40)	2.65 (1.17)
		2	4.67 (1.47)	5.17 (1.14)	5.82 (2.27)	3.97 (1.44)	5.82 (2.20)
		3	6.00 (0.68)	6.27 (0.99)	7.19 (0.72)	4.77 (0.80)	5.30 (1.75)
		4	11.93 (1.22)	12.68 (1.53)	14.88 (0.29)	9.31 (1.39)	9.88 (2.44)
	+ 4 cm	1	2.03 (0.55)	2.43 (0.45)	2.70 (0.55)	1.43 (0.34)	2.92 (1.30)
		2	4.45 (1.46)	4.74 (1.06)	5.28 (2.06)	3.30 (1.21)	6.45 (2.42)
		3	5.71 (0.66)	5.72 (0.85)	6.91 (0.59)	4.00 (0.70)	5.72 (2.06)
		4	11.24 (1.08)	11.67 (1.37)	13.58 (0.25)	7.85 (1.25)	10.73 (2.87)
	+ 6 cm	1	1.78 (0.49)	2.00 (0.37)	2.33 (0.48)	1.09 (0.24)	2.85 (1.30)
		2	3.93 (1.34)	3.98 (0.95)	4.41 (1.65)	2.51 (0.90)	6.39 (2.40)
		3	5.06 (0.63)	4.82 (0.70)	6.31 (0.42)	3.07 (0.51)	5.52 (2.10)
		4	9.79 (0.90)	9.87 (1.13)	11.80 (0.47)	6.05 (0.93)	10.35 (3.05)
Siemens SOMATOM Definition Flash	- 6 cm	1	0.75 (0.31)	0.71 (0.26)	1.23 (0.42)	0.63 (0.22)	1.07 (0.55)
		2	2.17 (0.69)	1.80 (0.43)	2.91 (1.17)	1.87 (0.54)	2.87 (1.15)
		3	2.73 (0.25)	2.26 (0.36)	3.55 (0.33)	2.45 (0.41)	2.59 (0.89)
		4	6.17 (0.79	5.14 (0.80)	8.35 (0.49)	4.84 (1.31)	5.31 (1.15)
	- 4 cm	1	0.80 (0.33)	0.81 (0.30)	1.27 (0.44)	0.66 (0.23)	1.19 (0.59)
		2	2.29 (0.72)	2.03 (0.48)	2.98 (1.20)	1.92 (0.54)	3.25 (1.28)
		3	2.89 (0.29)	2.54 (0.40)	3.73 (0.38)	2.51 (0.40)	2.93 (1.01)
		4	6.61 (0.81)	5.79 (0.85)	8.80 (0.56)	5.01 (1.31)	6.00 (1.35)
	- 2 cm	1	0.86 (0.35)	0.92 (0.34)	1.42 (0.50)	0.68 (0.24)	1.32 (0.64)
		2	2.46 (0.78)	2.33 (0.554)	3.35 (1.38)	2.00 (0.57)	3.65 (1.42)
		3	3.10 (0.31)	2.88 (0.46)	4.19 (0.41)	2.58 (0.43)	3.23 (1.14)
		4	7.11 (0.84)	6.55 (0.91)	9.87 (0.57)	5.19 (1.33)	6.72 (1.58)
	+ 2 cm	1	0.91 (0.37)	1.06 (0.38)	1.42 (0.50)	0.66 (0.22)	1.36 (0.65)
		2	2.57 (0.82)	2.70 (0.63)	3.35 (1.38)	1.91 (0.54)	3.88 (1.49)
		3	3.28 (0.35)	3.24 (0.51)	4.19 (0.41)	2.49 (0.42)	3.35 (1.33)
		4	7.53 (0.86)	7.52 (0.91)	9.87 (0.57)	5.09 (1.30)	7.18 (1.92)
	+ 4 cm	1	0.82 (0.33)	0.96 (0.34)	1.32 (0.47)	0.57 (0.19)	1.09 (0.51)
		2	2.32 (0.75)	2.49 (0.60)	3.10 (1.26)	1.64 (0.48)	3.22 (1.24)
		3	2.95 (0.33)	2.94 (0.44)	3.92 (0.39)	2.11 (0.33)	2.71 (1.10)
		4	6.80 (0.80)	6.89 (0.88)	9.03 (0.58)	4.36 (1.11)	6.00 (1.65)
	+ 6 cm	1	0.80 (0.31)	0.90 (0.32)	1.29 (0.45)	0.51 (0.17)	1.00 (0.46)

https://doi.org/10.3390/jimaging12030123

		2	2.24 (0.72)	2.37 (0.58)	3.00 (1.18)	1.48 (0.43)	2.97 (1.15)
		3	2.90 (0.35)	2.79 (0.42)	3.90 (0.39)	1.93 (0.30)	2.49 (1.11)
		4	6.57 (0.76)	6.56 (0.84)	8.73 (0.56)	3.97 (1.01)	5.46 (1.48)
Canon Aquilion ONE	- 6 cm	1	2.73 (0.57)	2.96 (0.44)	3.09 (0.45)	2.44 (0.47)	3.80 (1.79)
		2	6.36 (2.05)	5.71 (1.38)	6.73 (2.65)	6.07 (2.25)	8.42 (3.27)
		3	8.66 (0.94)	7.80 (1.19)	8.90 (0.81)	7.89 (1.14)	8.31 (2.63)
		4	14.58 (1.95)	12.89 (1.71)	15.78 (0.39)	12.73 (2.72)	12.96 (2.52)
	- 4 cm	1	2.96 (0.64)	3.30 (0.48)	3.33 (0.49)	2.55 (0.49)	4.11 (1.91)
		2	6.84 (2.22)	6.39 (1.55)	7.15 (2.80)	6.26 (2.30)	9.12 (3.50)
		3	9.30 (1.03)	8.67 (1.29)	9.50 (0.83)	8.09 (1.5)	8.99 (2.88)
		4	15.68 (1.98)	14.40 (1.83)	16.60 (0.32)	13.08 (2.67)	14.12 (2.84)
	- 2 cm	1	2.89 (0.63)	3.31 (0.48)	3.23 (0.48)	2.40 (0.45)	3.98 (1.84)
		2	6.68 (2.19)	6.49 (1.60)	6.96 (2.71)	5.86 (2.15)	8.91 (3.41)
		3	9.05 (1.03)	8.71 (1.28)	9.25 (0.78)	7.51 (1.04)	8.72 (2.84)
		4	15.34 (1.84)	14.60 (1.80)	16.06 (0.34)	12.19 (2.38)	13.84 (2.90)
	+ 2 cm	1	2.84 (0.64)	3.40 (0.47)	3.18 (0.49)	2.18 (0.39)	3.72 (1.69)
		2	6.50 (2.13)	6.77 (1.69)	6.81 (2.63)	5.19 (1.87)	8.45 (3.19)
		3	8.80 (1.08)	8.90 (1.27)	9.24 (0.80)	6.58 (0.91)	8.16 (2.79)
		4	15.00 (1.69)	15.21 (1.86)	15.74 (0.64)	10.77 (1.97)	13.16 (2.96)
	+ 4 cm	1	2.85 (0.65)	3.48 (0.48)	3.22 (0.52)	2.09 (0.37)	3.60 (1.64)
		2	6.51 (2.14)	7.00 (1.74)	6.94 (2.59)	4.95 (1.75)	8.22 (3.13)
		3	8.82 (1.14)	9.08 (1.30)	9.49 (0.97)	6.24 (0.86)	7.90 (2.79)
		4	15.10 (1.68)	15.69 (1.92)	16.04 (0.73)	10.26 (1.83)	12.86 (2.98)
	+ 6 cm	1	2.67 (0.62)	3.30 (0.46)	3.02 (0.52)	1.88 (0.32)	3.23 (1.48)
		2	6.08 (2.02)	6.69 (1.72)	6.54 (2.51)	4.37 (1.54)	7.47 (2.82)
		3	8.28 (1.13)	8.65 (1.23)	9.18 (1.03)	5.54 (0.77)	7.12 (2.63)
		4	14.24 (1.56)	15.12 (1.86)	15.43 (0.68)	9.17 (1.60)	11.68 (2.79)
Philips Spectral CT 7500	- 6 cm	1	1.98 (0.40)	2.04 (0.31)	2.47 (0.40)	1.89 (0.44)	2.94 (1.42)
		2	4.34 (1.37)	3.72 (0.84)	5.00 (1.86)	3.81 (1.58)	6.02 (2.33)
		3	5.90 (0.61)	5.11 (0.83)	6.83 (0.78)	5.41 (0.42)	5.74 (1.68)
		4	10.58 (1.29)	9.20 (1.24)	13.25 (0.78)	9.24 (2.30)	9.73 (2.04)
	- 4 cm	1	2.02 (0.42)	2.17 (0.33)	2.48 (0.38)	1.90 (0.44)	3.02 (1.44)
		2	4.41 (1.40)	3.96 (0.89)	5.00 (1.87)	3.78 (1.55)	6.22 (2.39)
		3	5.99 (0.63)	5.40 (0.88)	6.84 (0.78)	5.31 (0.38)	5.91 (1.74)
		4	10.79 (1.25)	9.74 (1.26)	13.30 (0.74)	9.12 (2.22)	10.13 (2.21)
	- 2 cm	1	2.23 (0.47)	2.49 (0.37)	2.74 (0.41)	2.01 (0.47)	3.23 (1.53)
		2	4.82 (1.54)	4.53 (1.05)	5.38 (2.04)	3.97 (1.62)	6.68 (2.54)
		3	6.52 (0.71)	6.18 (0.98)	7.46 (0.83)	5.55 (0.35)	6.33 (1.86)
		4	11.83 (1.34)	11.21 (1.52)	14.42 (0.93)	9.58 (2.30)	11.01 (2.53)
	+ 2 cm	1	2.60 (0.56)	3.13 (0.46)	3.27 (0.54)	2.23 (0.54)	3.29 (1.51)
		2	5.58 (1.76)	5.77 (1.31)	6.48 (2.39)	4.35 (1.76)	6.89 (2.57)
		3	7.55 (0.85)	7.57 (1.29)	8.66 (1.00)	6.08 (0.39)	6.53 (2.00)
		4	13.80 (1.50)	13.86 (1.73)	17.15 (.10)	10.61 (2.50)	11.52 (2.76)
	+ 4 cm	1	2.06 (0.44)	2.42 (0.34)	2.53 (0.41)	1.68 (0.41)	2.74 (1.24)
		2	4.43 (1.43)	4.54 (1.08)	5.09 (1.89)	3.28 (1.30)	5.78 (2.14)
		3	6.06 (0.70)	6.00 (0.98)	7.13 (0.83)	4.58 (0.28)	5.41 (1.70)
		4	10.98 (1.18)	11.03 (1.41)	13.50 (0.74)	7.96 (1.88)	9.57 (2.41)
	+ 6 cm	1	1.95 (0.42)	2.31 (0.33)	2.46 (0.42)	1.54 (0.38)	2.46 (1.13)
		2	4.20 (1.35)	4.37 (1.05)	4.95 (1.81)	3.01 (1.18)	5.23 (1.92)
		3	5.75 (0.71)	5.72 (0.96)	6.99 (0.87)	4.20 (0.26)	4.85 (1.59)
		4	10.40 (1.12)	10.57 (1.36)	13.15 (0.80)	7.31 (1.72)	8.63 (2.24)

Absolute organ doses | Horizontal off-centring with dual localiser			
**Scanner**	**Position**	**BMI class**	**Organ**

	deviation		Lung	Heart	Breast	Liver	Thyroid
GE Revolution CT	- 6 cm	1	1.52 (0.28)	1.74 (0.26)	2.29 (0.36)	1.57 (0.33)	1.60 (0.79)
		2	3.58 (1.08)	3.44 (0.77)	4.90 (1.99)	4.12 (1.53)	3.58 (1.41)
		3	4.58 (0.47)	4.27 (0.69)	5.42 (0.40)	5.06 (0.91)	3.41 (1.04)
		4	9.06 (1.19)	8.35 (1.22)	12.05 (1.07)	9.50 (1.51)	6.12 (1.25)
	- 4 cm	1	1.30 (0.24)	1.71 (0.28)	1.85 (0.41)	1.56 (0.32)	1.38 (0.68)
		2	3.00 (0.84)	3.35 (0.72)	4.40 (1.89)	4.00 (1.47)	3.11 (1.21)
		3	3.84 (0.43)	4.01 (0.69)	4.44 (0.46)	4.81 (0.85)	2.92 (0.90)
		4	7.59 (1.00)	7.89 (1.20)	10.37 (1.20)	9.04 (1.40)	5.30 (1.12)
	- 2 cm	1	1.84 (0.38)	2.16 (0.30)	2.57 (0.30)	1.56 (0.32)	2.27 (1.11)
		2	4.39 (1.40)	4.40 (0.98)	5.36 (2.09)	4.02 (1.47)	5.19 (1.99)
		3	5.56 (0.56)	5.46 (0.81)	6.40 (0.38)	4.86 (0.84)	4.82 (1.52)
		4	11.11 (1.24)	10.81 (1.32)	13.62 (0.67)	9.38 (1.37)	8.82 (1.97)
	+ 2 cm	1	1.80 (0.39)	2.21 (0.29)	2.35 (0.31)	1.41 (0.28)	2.60 (1.32)
		2	4.31 (1.40)	4.62 (1.04)	5.12 (2.02)	3.56 (1.29)	6.05 (2.26)
		3	5.52 (0.63)	5.61 (0.86)	6.46 (0.51)	4.28 (0.73)	5.45 (1.81)
		4	10.90 (1.08)	11.28 (1.32)	13.03 (0.49)	8.31 (1.23)	10.12 (2.66)
	+ 4 cm	1	1.43 (0.29)	2.00 (0.28)	1.99 (0.40)	1.29 (0.25)	1.52 (0.73)
		2	3.30 (0.98)	4.13 (0.93)	4.54 (1.85)	3.21 (1.15)	3.59 (1.35)
		3	4.26 (0.53)	4.79 (0.72)	5.08 (0.49)	3.85 (0.64)	3.24 (1.12)
		4	8.50 (0.91)	9.69 (1.28)	10.90 (0.95)	7.44 (1.14)	6.14 (1.58)
	+ 6 cm	1	1.46 (0.29)	2.02 (0.27)	2.15 (0.38)	1.20 (0.23)	1.37 (0.66)
		2	3.35 (1.04)	4.19 (1.00)	4.59 (1.86)	2.97 (1.06)	3.31 (1.24)
		3	4.33 (0.57)	4.79 (0.65)	5.30 (0.41)	3.59 (0.59)	2.96 (1.08)
		4	8.64 (0.94)	9.80 (1.34	11.16 (1.00)	6.95 (1.11)	5.68 (1.47)
Siemens SOMATOM Definition Flash	- 6 cm	1	0.71 (0.14)	0.69 (0.10)	1.07 (0.15)	0.63 (0.16)	1.01 (0.54)
		2	2.39 (0.74)	2.04 (0.47)	3.27 (1.27)	2.14 (0.60)	3.06 (1.21)
		3	3.01 (0.28)	2.55 (0.42)	3.93 (0.40)	2.82 (0.47)	2.78 (0.94)
		4	6.86 (0.86)	5.81 (0.91)	9.31 (0.65)	5.59 (1.52)	5.73 (1.22)
	- 4 cm	1	0.89 (0.18)	0.95 (0.14)	1.37 (0.19)	0.79 (0.20)	1.35 (0.73)
		2	3.01 (0.95)	2.78 (0.65)	4.13 (1.68)	2.66 (0.75)	4.01 (1.55)
		3	3.78 (0.37)	3.44 (0.55)	4.89 (0.51)	3.46 (0.58)	3.68 (1.16)
		4	8.77 (1.09)	7.89 (1.16)	12.02 (0.73)	6.92 (1.82)	7.71 (1.83)
	- 2 cm	1	0.90 (0.19)	0.98 (0.15)	1.31 (0.17)	0.70 (0.18)	1.43 (0.75)
		2	3.03 (0.96)	2.88 (0.66)	3.91 (1.61)	2.36 (0.67)	4.45 (1.73)
		3	3.80 (0.37)	3.56 (0.56)	4.89 (0.47)	3.08 (0.53)	3.93 (1.34)
		4	8.78 (1.06)	8.14 (1.15)	11.61 (0.74)	6.21 (1.62)	8.25 (1.96)
	+ 2 cm	1	0.79 (0.17)	0.96 (0.13)	1.21 (0.15)	0.56 (0.14)	1.07 (0.54)
		2	2.64 (0.83)	2.86 (0.64)	3.57 (1.43)	1.85 (0.53)	3.50 (1.35)
		3	3.35 (0.36)	3.46 (0.54)	4.45 (0.47)	2.40 (0.39)	3.00 (1.16)
		4	7.78 (0.90)	7.92 (1.08)	10.44 (0.88)	4.96 (1.30)	6.53 (1.68)
	+ 4 cm	1	0.69 (0.15)	0.82 (0.11)	1.02 (0.13)	0.49 (0.12)	0.97 (0.50)
		2	2.31 (0.74)	2.48 (0.59)	3.08 (1.24)	1.64 (0.47)	3.20 (1.23)
		3	2.95 (0.32)	2.94 (0.44)	3.92 (038)	2.11 (0.33)	2.70 (1.09)
		4	6.76 (0.80)	6.86 (0.90)	8.97 (0.59)	4.35 (1.12)	5.95 (1.65)
	+ 6 cm	1	0.57 (0.12)	0.67 (0.09)	0.87 (0.12)	0.39 (0.10)	0.72 (0.37)
		2	1.90 (0.61)	2.06 (0.51)	2.61 (1.02)	1.29 (0.37)	2.43 (0.94)
		3	2.45 (0.30)	2.41 (0.37)	3.36 (0.36)	1.68 (0.27)	2.04 (0.87)
		4	5.59 (0.64)	5.69 (0.73)	7.61 (0.48)	3.46 (0.88)	4.54 (1.27)
Canon Aq-uilion ONE	- 6 cm	1	2.63 (0.55)	2.84 (0.42)	2.96 (0.44)	2.35 (0.46)	3.66 (1.72)
		2	6.11 (1.98)	5.49 (1.33)	6.49 (2.56)	5.83 (2.17)	8.09 (3.13)
		3	8.31 (0.90)	7.49 (1.14)	8.55 (0.80)	7.58 (1.09)	7.96 (2.53)
		4	14.00 (1.87)	12.38 (1.63)	15.16 (0.37)	12.22 (2.63)	12.46 (2.45)

	- 4 cm	1	2.87 (0.62)	3.19 (0.46)	3.22 (0.47)	2.47 (0.47)	3.98 (1.85)
		2	6.66 (2.18)	6.22 (1.52)	6.99 (2.74)	6.10 (2.25)	8.87 (3.41)
		3	9.00 (1.00)	8.39 (1.26)	9.17 (0.81)	7.84 (1.11)	8.71 (2.80)
		4	15.20 (1.92)	13.96 (1.77)	16.09 (0.31)	12.68 (2.59)	13.72 (2.76)
	- 2 cm	1	2.92 (0.64)	3.34 (0.48)	3.25 (0.49)	2.42 (0.45)	4.02 (1.85)
		2	6.73 (2.18)	6.54 (1.58)	7.02 (2.71)	5.90 (2.14)	8.96 (3.41)
		3	9.11 (1.04)	8.76 (1.29)	9.29 (0.78)	7.56 (1.06)	8.78 (2.90)
		4	15.43 (1.85)	14.69 (1.82)	16.14 (0.39)	12.26 (2.40)	13.93 (2.91)
	+ 2 cm	1	2.73 (0.62)	3.27 (0.46)	3.06 (0.48)	2.09 (0.38)	3.58 (1.63)
		2	6.26 (2.07)	6.52 (1.63)	6.58 (2.55)	5.01 (1.81)	8.12 (3.07)
		3	8.47 (1.04)	8.57 (1.23)	8.89 (0.80)	6.33 (0.88)	7.86 (2.69)
		4	13.94 (2.23)	14.14 (2.40)	14.02 (1.79)	10.01 (2.21)	12.24 (3.21)
	+ 4 cm	1	2.78 (0.63)	3.39 (0.46)	3.14 (0.49)	2.04 (0.36)	3.51 (1.60)
		2	6.35 (2.10)	6.81 (1.73)	6.73 (2.58)	4.82 (1.73)	8.05 (3.04)
		3	8.56 (1.13)	8.82 (1.28)	9.17 (0.93)	6.06 (0.85)	7.69 (2.75)
		4	14.71 (1.63)	15.30 (1.87)	15.64 (0.71)	10.00 (1.78)	12.53 (2.91)
	+ 6 cm	1	2.58 (0.59)	3.19 (0.43)	2.94 (0.48)	1.81 (0.31)	3.12 (1.42)
		2	5.85 (1.94)	6.45 (1.66)	6.31 (2.43)	4.21 (1.50)	7.20 (2.72)
		3	7.97 (1.08)	8.32 (1.18)	8.86 (0.99)	5.33 (0.73)	6.85 (2.50)
		4	13.67 (1.52)	14.51 (1.81)	14.82 (0.67)	8.80 (1.55)	11.22 (2.70)
Philips Spectral CT 7500	- 6 cm	1	2.37 (0.48)	2.43 (0.36)	2.94 (0.46)	2.23 (0.53)	3.53 (1.68)
		2	5.18 (1.64)	4.41 (1.00)	5.93 (2.21)	4.51 (1.86)	7.22 (2.77)
		3	7.06 (0.73)	6.09 (0.99)	8.14 (0.94)	6.42 (0.50)	6.88 (2.01-
		4	12.64 (1.53)	10.94 (1.46)	15.71 (0.93)	10.95 (2.73)	11.66 (2.44)
	- 4 cm	1	2.34 (0.48)	2.45 (0.37)	2.79 (0.42)	2.10 (0.50)	3.46 (1.61)
		2	5.08 (1.62)	4.46 (1.01)	5.62 (2.09)	4.22 (1.73)	7.07 (2.69)
		3	6.92 (0.73)	6.14 (0.99)	7.90 (0.89)	5.94 (0.42)	6.75 (1.98)
		4	12.46 (1.46)	11.07 (1.46)	15.08 (0.81)	10.20 (2.48)	11.56 (2.48)
	- 2 cm	1	2.50 (0.52)	2.74 (0.41)	2.95 (0.44)	2.17 (0.53)	3.42 (1.57)
		2	5.40 (1.71)	5.03 (1.11)	6.00 (2.18)	4.26 (1.76)	7.07 (2.68)
		3	7.34 (0.80)	6.81 (1.16)	8.25 (0.94)	6.08 (0.43)	6.75 (2.04)
		4	13.24 (1.50)	12.27 (1.52)	16.06 (0.76)	10.51 (2.61)	11.68 (2.59)
	+ 2 cm	1	2.67 (0.56)	3.14 (0.45)	3.24 (0.51)	2.19 (0.56)	3.39 (1.50)
		2	5.70 (1.81)	5.80 (1.34)	6.47 (2.36)	4.17 (1.70)	7.07 (2.63)
		3	7.75 (0.89)	7.67 (1.30)	8.86 (1.01)	5.92 (0.42)	6.67 (2.05)
		4	14.11 (1.52)	13.96 (1.76)	17.26 (0.95)	10.32 (2.56)	11.75 (2.76)
	+ 4 cm	1	2.34 (0.49)	2.70 (0.38)	2.79 (0.41)	1.80 (0.44)	3.00 (1.34)
		2	5.02 (1.62)	5.04 (1.17)	5.62 (2.03)	3.53 (1.40)	6.30 (2.33)
		3	6.86 (0.78)	6.69 (1.14)	8.07 (0.95)	4.95 (0.31)	5.90 (1.85)
		4	12.44 (1.31)	12.30 (1.53)	15.13 (0.67)	8.64 (2.02)	10.45 (2.59)
	+ 6 cm	1	2.34 (0.50)	2.83 (0.40)	2.95 (0.51)	1.86 (0.47)	2.87 (1.31)
		2	5.02 (1.59)	5.32 (1.26)	6.03 (2.17)	3.62 (1.44)	6.12 (2.24)
		3	6.86 (0.84)	6.89 (1.18)	8.26 (1.03)	5.08 (0.33)	5.66 (1.83)
		4	12.44 (1.35)	12.73 (1.62)	15.83 (0.91)	8.83 (2.08)	10.11 (2.62)
